# Nonlinear Rayleigh wave propagation in a three-layer sandwich structure in dual-phase-lag

**DOI:** 10.1038/s41598-024-73912-9

**Published:** 2024-11-07

**Authors:** A. A. Youssef, N. K. Amein, F. A. Salama, A. F. Ghaleb, Ethar A. A. Ahmed

**Affiliations:** 1 Department of Mathematics, Faculty of Science, Suez Canal University, Ismailia, Egypt; 2 Department of Mathematics, Faculty of Science, Cairo University, Giza, 12613 Egypt; 3 School of Engineering and Applied Sciences, Nile University, Giza, 12588 Egypt

**Keywords:** Plane thermoelasticity, Nonlinear Rayleigh waves, Three-layer sandwich structure, Dual-phase-lag, Temperature-dependent material properties, Applied mathematics, Statistical physics, thermodynamics and nonlinear dynamics

## Abstract

Plane, nonlinear Rayleigh wave propagation is investigated in a three-layer sandwich structure of a thermoelastic medium, within the frame of dual-phase-lag theory. The thermal conductivity is taken as a linear function of temperature. This induces nonlinearity in the evolution equations for the heat flux components. A particular solution is found in the form of Poincaré expansion in a small parameter that reflects the fluctuations of temperature about a steady value. This solution is discussed and plots are provided for a special case when both external faces of the structure are traction-free and under prescribed temperature regime. It is noted, in particular, that some quantities of practical interest suffer jumps at the interfaces. Some of the jumps appear only starting from the second order of approximation. The existence of jumps may be favourably used to carry out some measurements of material parameters.

## Introduction

Sandwich structures are the subject of many applications in virtually every branch of industry. Plate structures have a wide range of applications in engineering disciplines, such as aerospace and mechanical engineering. The advantages of layered media over conventional materials are that these composites can be designed to have a low overall density, a high strength-to-weight ratio, and a high stiffness-to-weight ratio. Good thermal, acoustical and other specific properties like electrical conductivity can also be achieved. This has boosted the development and use of layered composites in the past few decades or so. A review of modern trends in theoretical developments, novel designs and modern applications of sandwich structures may be found in^1^.

The utilization of these structures in aerospace applications is extensive due to their capacity to deliver a high strength-to-weight ratio, impact resistance, and structural stability^2^. Various core materials, including honeycomb, foam, and metallic structures, are incorporated in the design of these structures, along with face sheets composed of materials such as carbon fiber and aluminum^3,4^. The manufacturing techniques employed for sandwich structures encompass processes like hand lay-up, autoclave application, and other advanced methods, rendering them well-suited for the demands of the aerospace industry^5,6^. Furthermore, sandwich structures find application in aircraft flaps, where optimized designs featuring trapezoidal corrugated cores or bio-inspired spiderweb cores enhance stiffness and alleviate stress levels, thus demonstrating their versatility and suitability in aerospace engineering applications.

Sandwich structures play a pivotal role in the realm of automotive engineering, providing notable benefits including elevated flexural stiffness, an advantageous strength-to-weight ratio, weight reduction, and decreased power consumption^3^. The incorporation of sandwich composites in the automotive sector is subjected to meticulous examination through tests focusing on quasi-static and fatigue loading, aiming to comprehend failure modes and damage features, which in turn assist in the selection of materials for components like automotive leaf springs^7^. Through the application of cutting-edge manufacturing methodologies like resin transfer molding, sandwich structures featuring carbon fiber surface layers and Kevlar honeycomb cores have been engineered to boost load-bearing capacities while upholding a lightweight nature^8^. These breakthroughs in sandwich composite materials make substantial contributions towards enhancing the structural performance of automotive vehicles, all the while ensuring optimal weight distribution and durability.

In many cases of practical importance, especially at nanoscale, it is necessary to take in consideration temperature effects. The dependence on temperature of the various material constants plays a fundamental role in the thermo-electromagneto-mechanical coupling analysis of solids. The dependence of thermal conductivity on temperature may be considered as an empirical relationship based on pure experimental data. Working within extended thermodynamics, not only provides a more natural framework for such complex structured media, but also allows to use the propagation of heat waves for the purposes of nondestructive testing and estimation of material parameters.

Lianzhi et al.^9^ investigated two-dimensional quasicrystal, simply supported nanoplates subjected to a temperature change on their top surface. They proposed a three-dimensional thermo-elastic analytical solution for such structures using nonlocal theory and pseudo-Stroh formalism. Sur and Kanoria^10^ investigated linear thermoelastic interactions in a three-dimensional homogeneous and isotropic sandwich structure under dual-phase-lag using normal mode analysis. Ahmed et al.^11^ investigated linear, thermoelastic wave propagation in a layered piezoelectric material composed of a slab bonded to a half-space of a dissimilar material, within dual-phase-lag model.

Yadav^12,13^ examined the behavior and propagation of waves within materials subjected to the influences of magnetic and thermal fields. The study analyzed the reflection characteristics of magneto-photothermal plasma waves in diffusive semiconductors, within the framework of two-temperature theory and multi-phase-lag thermoelasticity. Furthermore, Yadav^14^ investigated the reflection of plane waves within a rotating triclinic solid half-space employing a fractional-order generalized magneto-thermoelasticity model. Singh and Yadav^15^ investigated the propagation attributes of thermoelastic Rayleigh waves in a transversely isotropic solid half-space subjected to initial stresses and a magnetic field.

Mandi et al.^16^ investigated analytically the Rayleigh wave generation in a stratified structure composed of a layer constrained between two half-spaces to analyze the effect of various material parameters. The propagation of nonlinear waves has attracted a great deal of attention in the past few years because of many important applications. Different theoretical approaches have been proposed to tackle problems of nonlinear elastic wave propagation in complex structured media, for example granular materials and phononic crystals. One may refer here to the incremental harmonic balance method^17^ that is an efficient replacement of the traditional perturbation methods for treating problems involving materials with strongly nonlinear periodic structures. Such techniques are basically related to nonlocal theories of Continuum Mechanics and are expected to explain a broader spectrum of phenomena. Vattré and Pan^18^ presented three-dimensional exact solutions by Fourier series expansions for temperature and thermoelastic stresses in multilayered orthotropic bonded, dissimilar, linearly thermoelastic, and rectangular plates under general boundary conditions. An improved Stroh formalism is formulated to include the thermal coupling with the Eringen nonlocal elasticity theory to capture small scale effects. Ahmed et al.^19^ produced an exact solution in closed form for a two-dimensional problem of heat conduction in a rigid thermal conductor within dual-phase-lag by one-sided Fourier transform. The effect of both relaxation times was put in evidence. Palermo et al.^20^ investigated, both analytically and numerically by finite elements, the propagation of Rayleigh waves in a half-space coupled to a nonlinear metasurface and obtained closed-form expressions to predict the dispersive characteristics of nonlinear Rayleigh waves. Hilal^21^ studied the problem of rotation of a linear and homogeneous microelongated thermoelastic medium within G-N theory of type III using Fourier and Laplace transforms. Ahmed and Abou-Dina^22^ deal with dual-phase-lag model within the generalized piezothermoelasticity, in an infinite uniform strip of finite width, by applying the normal mode analysis. A state-of-the-art review on the mechanical properties of sandwich structures and the effect of temperature variation on these properties may be found in^23^. Gartsev et al.^24^ proposed a numerical and experimental investigation of nonlinear interaction of Rayleigh waves in isotropic materials. Nonlinear Rayleigh wave propagation in generalized thermoelastic media with temperature-dependent thermal conductivity was considered by Youssef et al.^25,26^. Extensive literature may be found therein. Peng and Pan^27^ study the size-dependent model of functionally graded sandwich microbeam resonators by nonlocal dual-phase-lag heat conduction and coupled stress theory. It is thus seen that nonlinear wave propagation in thermoelasticity is usually tackled by numerical techniques due to their complexity.

It is the purpose of the present work to extend the scope of previous work in^25,26^ to investigate nonlinear Rayleigh wave propagation in three-layer sandwich structures of thermoelastic materials with temperature dependent thermal conductivity. A particular solution is obtained in the form of Poincaré small parameter expansion. This solution is discussed and plots are provided. As already noted in previously cited work, some quantities have finite discontinuities at the interfaces, while others are continuous there. Jumps arise naturally as a result of a correct application of interface conditions, deduced from the field equations as is usually done in Continuum Mechanics. This fact may be favourably used to carry out some measurements. For the sake of conciseness, details of calculations will be omitted, as these may be found in the cited references.

## Problem formulation

**Fig. 1:**
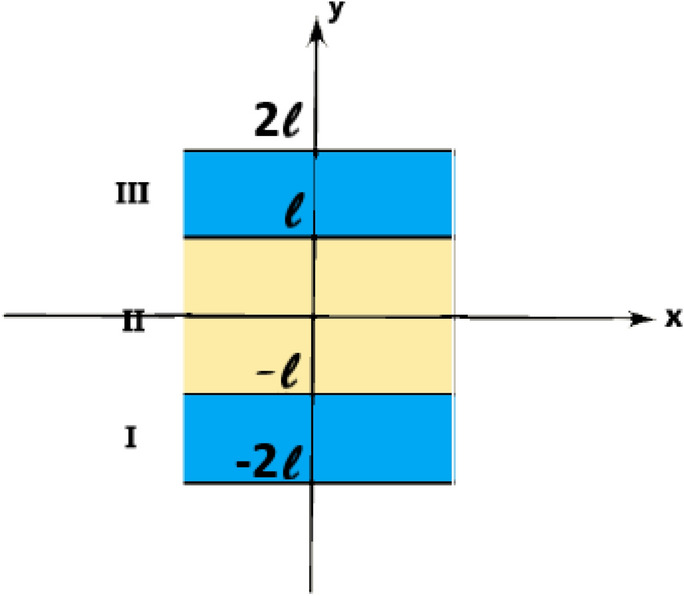
Geometry of the problem.

We investigate 2D nonlinear Rayleigh wave propagation in a three-layer sandwich structure of transversely isotropic thermoelastic materials, with temperature-dependent thermal conductivities. A system of orthogonal Cartesian coordinates (*x*, *y*, *z*) is used for the description of the problem, the *y*-coordinate being directed along the normal to the parallel bounding surfaces of the materials, as shown in Fig. 1, the layers are labelled I, II and III. For the sake of concreteness, the central layer is taken to have thickness $2 \ell$, while the other two layers have thickness $\ell$, the structure is positioned symmetric w.r.to the *y*-axis. There is no dependence of the solution on the *z*-coordinate.

## Basic equations

The following equations take place individually in each of the three layers forming the structure under investigation. The labels characterizing the different layers will appear only when dealing with the boundary conditions at the external boundaries, and the interfacial conditions.

The eight basic unknown functions of the problem in each of the layers are: $v_x,v_y$- the velocity components, $\sigma _{xx}, \sigma _{yy}, \sigma _{xy}$-the identically non-vanishing stress components, $\theta$- the temperature as measured from a reference temperature $\theta _0$ and $q_x, q_y$- the heat flux components. All other stress components vanish identically. The relaxation times have been taken the same for both spatial directions for simplicity. Having solved the problem, the mechanical displacement components $u_x,u_y$ may be obtained from the velocities through the relations:

1 $$\begin{aligned} v_x= & \frac{\partial u_x}{\partial t}, \end{aligned}$$

2 $$\begin{aligned} v_y= & \frac{\partial u_y}{\partial t}. \end{aligned}$$

by integration w.r.t. time. Body forces and heat sources are disregarded. The system of two-dimensional dimensionless equations of plane thermoelasticity for a transversely isotropic material within the theory of extended thermodynamics to be considered is given in^25,26^. It involves two equations of motion, the equation of energy, two evolution laws for the heat flux components and three generalized constitutive relations for the stresses (Hooke’s laws), differentiated w.r.t. time. More details may be found in the two cited references. The characteristic quantities for temperature, length, time and heat flux used in the dimension analysis are:

$$\begin{aligned} \Theta _{0}=\theta _{0}, \quad L_{0}= \sqrt{\frac{\tau _0 K_0}{\rho C_e}}, \quad T_0 = \tau _0, \quad Q_{0}=\Theta _{0} \sqrt{\frac{\rho C_e K_0 }{\tau _0 }}, \end{aligned}$$

by which it is seen that the characteristic velocity

$$\begin{aligned} \frac{L_0}{T_0} = \sqrt{\frac{K_0}{\rho C_e}} \, \frac{1}{\sqrt{\tau _0}} \end{aligned}$$

is closely related to the velocity of second sound. Here, $\rho$ denotes the mass density, $K_0$- a characteristic heat conductivity, $C_e$- the specific heat and $\tau _0$- a characteristic thermal relaxation time.

The system of equations reads^25^:

3 $$\begin{aligned} \frac{\partial v_x}{\partial t}-\beta _1\left( \frac{\partial \sigma _{xx}}{\partial x}-\beta _4\frac{\partial \theta }{\partial x}+\frac{\partial \sigma _{xy}}{\partial y}\right)= & 0, \end{aligned}$$

4 $$\begin{aligned} \frac{\partial v_y}{\partial t}-\beta _1\left( \frac{\partial \sigma _{xy}}{\partial x}-\beta _4\frac{\partial \theta }{\partial y}+\frac{\partial \sigma _{yy}}{\partial y}\right)= & 0, \end{aligned}$$

5 $$\begin{aligned} \frac{\partial \theta }{\partial t}+\beta _2\left( \frac{\partial v_x}{\partial x}+\frac{\partial v_y}{\partial y}\right) +\left( \frac{\partial \, q_x}{\partial x}+\frac{\partial \, q_y}{\partial y}\right)= & 0, \end{aligned}$$

6 $$\begin{aligned} \tau _q\frac{\partial q_x}{\partial t}+q_x+K_{11}(\theta ) \left( \frac{\partial \theta }{\partial x}+ \tau _{\theta } \frac{\partial ^2\theta }{\partial x\partial t}\right)= & 0, \end{aligned}$$

7 $$\begin{aligned} \tau _q\frac{\partial q_y}{\partial t}+q_y+K_{22}(\theta )\left( \frac{\partial \theta }{\partial y}+ \tau _{\theta } \frac{\partial ^2\theta }{\partial y\partial t}\right)= & 0, \end{aligned}$$

8 $$\begin{aligned} \frac{\partial }{\partial t}\left( \beta \sigma _{xx}-\alpha \sigma _{yy}\right) -\frac{\partial v_x}{\partial x}= & 0, \end{aligned}$$

9 $$\begin{aligned} \frac{\partial }{\partial t}\left( -\alpha \sigma _{xx}+\beta \sigma _{yy}\right) -\frac{\partial v_y}{\partial y}= & 0, \end{aligned}$$

10 $$\begin{aligned} \frac{\partial \sigma _{xy}}{\partial t}-\frac{\partial v_x}{\partial y}-\frac{\partial v_y}{\partial x}= & 0, \end{aligned}$$

with dimensionless coefficients:

$$\begin{aligned} \beta _1 = \frac{\mu \tau _0 C_e}{K_0}, \quad \beta _2 = \frac{\gamma }{\rho C_e}, \quad \beta _3 = \frac{\lambda }{\mu }, \quad \beta _4 = \frac{\gamma \theta _0}{\mu }, \end{aligned}$$

and

$$\begin{aligned} \alpha = \frac{\lambda }{4 ( \lambda + \mu )}, \qquad \beta = \frac{\lambda + 2 \mu }{4 ( \lambda + \mu )}. \end{aligned}$$

Here, $\rho$ is the mass density, $\lambda , \mu$- Lam$\acute{e}$ coefficients, $\gamma$- the thermoelastic coefficient, $K_{11}, K_{22}$- the temperature-dependent coefficients of heat conduction and $\tau _q, \tau _{\theta }$- the relaxation times related to temperature and heat flux respectively. Nonlinearity appears only in the evolution equations for heat flux (6) and (7) through the dependence on temperature of the heat conduction coefficients.

### Particular solution

For nonlinearities of the type explained above and shown in the evolution equations for heat flux, we look for a particular solution for all the unknowns of the problem to describe plane, nonlinear Rayleigh wave propagation along the *x*-direction, for which the amplitude decreases exponentially in depth into the medium, in the form of usual Poincaré expansions in a small parameter $\varepsilon$, say. In what follows, only the first two orders of approximation will be considered. The approximate solution takes the form:

11 $$\begin{aligned} & \{ v_x, v_y, \theta , \sigma _{xx}, \sigma _{yy}, \sigma _{xy}, q_x, q_y \}(x,y,t) = \nonumber \\ & \quad \varepsilon \{ v^*_x, v^*_y, \theta ^*, \sigma ^*_{xx}, \sigma ^*_{yy}, \sigma ^*_{xy}, q^*_x, q^*_y \}(y) \, e^{i(k x-\omega _i t) - \omega _r t} + \nonumber \\ & \quad + \varepsilon ^2 \{ v^{**}_x, v^{**}_y, \theta ^{**}, \sigma ^{**}_{xx}, \sigma ^{**}_{yy}, \sigma ^{**}_{xy}, q^{**}_x, q^{**}_y \}(y) \, e^{2i(k x - \omega _i t) -2 \omega _r t} + \cdots , \end{aligned}$$

where the “starred”  and the “double-starred”  quantities denote the amplitudes of the functions at the first and the second approximations respectively, and $\varepsilon$ is an adequately chosen positive small parameter representing the amplitude of variation of the temperature as measured from the reference temperature $\Theta _0$. Parameter *k* denotes the wavenumber, $\omega _r$ is the frequency and $\omega _i$- the time damping, or attenuation coefficient. The dependence of some of the unknowns on the others is taken into account in the forthcoming relations. For present considerations, the normal mechanical load and the temperature are prescribed at the boundaries outer boundaries of the structure. the solution will have selected wave number, frequency and attenuationg coefficient, rather than taking in account the dispersion relation. Our aim is to assess the influence on surface wave propagation of the linear dependence of the heat conduction coefficient on temperature, as well as thermal relaxation times.

Substituting from Eq. (11) into the basic Eqs. (3)–(10) and denoting $D= \frac{d}{dy}$, one obtains a system of homogeneous linear ordinary differential equations of the first order, and a system of non-homogeneous linear ordinary differential equations of the first order, in the first two orders of approximation respectively. Here:

12 $$\begin{aligned} K_{11} \left( \theta \right) =K_{22} \left( \theta \right) =K_{0} (1+\eta \, \theta ), \end{aligned}$$

expressing a linear dependence of the heat conduction coefficients on temperature in any of the three layers. Such a dependence may be relevant, especially at high temperatures when the material characteristics can no longer be treated as constants. We assume that parameter $\eta$ is the same for all the layers, and moreover has an order of magnitude equal to unity. As explained in^26^, the small parameter $\varepsilon$, is taken as a measure of the amplitude of the propagating linear waves. The wave amplitude of the nonlinear component is $\varepsilon ^2$, and its contribution is therefore expected to be much smaller than that of the linear wave. The present investigation focuses on the role of parameter $\eta$ on the behavior of the solution under Rayleigh wave propagation.

### Governing equations at the first two orders of approximation

The system of homogeneous linear differential equations of the first order:

13 $$\begin{aligned} D v ^*_x= & A_1 \, v ^*_y +A_2 \, \sigma _{xy}^*, \end{aligned}$$

14 $$\begin{aligned} D v ^*_y= & A_{3} \, v ^*_x +A_4 \, \sigma ^*_{yy}, \end{aligned}$$

15 $$\begin{aligned} D \theta ^*= & A_5 \, q^*_y, \end{aligned}$$

16 $$\begin{aligned} D \sigma ^*_{yy}= & A_{6} \, v ^*_y +A_1 \, \sigma ^*_{xy} +A_7 \, q^*_y, \end{aligned}$$

17 $$\begin{aligned} D \sigma ^*_{xy}= & A_8 \, v ^*_x + A_9 \, \theta ^*+A_3\sigma ^*_{yy}, \end{aligned}$$

18 $$\begin{aligned} D q^*_y= & A_{10} \, \theta ^*+A_{11} \, v ^*_x +A_{12} \, \sigma ^*_{yy} , \end{aligned}$$

together with

19 $$\begin{aligned} \sigma ^*_{xx}= & A_{13} \, v ^*_x +A_{14 } \, \sigma ^*_{yy}, \end{aligned}$$

20 $$\begin{aligned} q^*_x= & A_{15 } \, \theta ^* . \end{aligned}$$

The system of non-homogeneous linear differential equations of the first order:

21 $$\begin{aligned} D v ^{**}_x= & A_{16} \, v ^{**}_y +A_{17} \, \sigma ^{**}_{xy}, \end{aligned}$$

22 $$\begin{aligned} D v ^{**}_y= & A_{18} \, v ^{**}_x + A_{19} \, \sigma ^{**}_{yy}, \end{aligned}$$

23 $$\begin{aligned} D \theta ^{**}= & A_{20} \, q^{**}_y +A_{31} \, \theta ^* D \theta ^*, \end{aligned}$$

24 $$\begin{aligned} D \sigma ^{**}_{yy}= & A_{21} \, v ^{**}_y +A_{16} \, \sigma ^{**}_{xy} +A_{22}q^{**}_y +A_{32} \theta ^* D \theta ^*, \end{aligned}$$

25 $$\begin{aligned} D \sigma ^{**}_{xy}= & A_{23} \, v ^{**}_x +A_{24} \, \theta ^{**}+A_{18} \, \sigma ^{**}_{yy}, \end{aligned}$$

26 $$\begin{aligned} D q^{**}_y= & A_{25 } \, \theta ^{**}+A_{26} \, v ^{**}_x +A_{27} \, \sigma ^{**}_{yy} + A_{33} \, \theta ^{*2} , \end{aligned}$$

together with

27 $$\begin{aligned} \sigma ^{**}_{xx}= & A_{28} v ^{**}_x +A_{29}\sigma ^{**}_{yy}, \end{aligned}$$

28 $$\begin{aligned} q^{**}_x= & A_{30}\theta ^{**}+A_{34} \theta ^{*2}. \end{aligned}$$

and $A_{j},j=1,2,\ldots ,34$ are constants listed in Appendix A. It clearly appears that quadratic expressions in $\theta ^*$ will be responsible for the generation of the solution at the second order of approximation.

## The homogeneous system

29 $$\begin{aligned} {\left( \begin{array}{c} D v_x^* \\ D v_y^* \\ D \theta ^* \\ D \sigma _{yy}^* \\ D \sigma _{xy}^* \\ D q_y^* \\ \end{array} \right) =\left( \begin{array}{cccccc} 0 & A_1 & 0 & 0 & A_2 & 0 \\ A_3 & 0 & 0 & A_4 & 0 & 0 \\ 0 & 0 & 0 & 0 & 0 & A_5 \\ 0 & A_6 & 0 & 0 & A_1 & A_7 \\ A_8 & 0 & A_9 & A_3 & 0 & 0 \\ A_{10} & 0 & A_{11} & A_{12} & 0 & 0 \\ \end{array} \right) \left( \begin{array}{c} v_x^* \\ v_y^* \\ \theta ^* \\ \sigma _{yy}^* \\ \sigma _{xy}^* \\ q_y^* \\ \end{array} \right) .} \end{aligned}$$

Assuming a solution of the form $e^{\xi y}$, the characteristic equation for the eigenvalues $\xi$ for this system of equations is obtained as:

30 $$\begin{aligned} \xi ^{6} - A \xi ^{4} + B \xi ^{2} - C = 0, \end{aligned}$$

where *A*, *B* and *C* are constants given by Mathematica 14.0.

Only three roots of this equation, $\xi _{n}, n=1,2,3$ with positive real parts, will contribute to the bounded solution. Following Eq.(30), any imaginary part of $\xi$ will result in a circular function of sine or cosine in the solution, i.e. an amplitude that is oscillating in y while damped exponentially in y. In the end, only the real part of the solution will have physical meaning. The solution of the system of Eq. (29) may be written conveniently in the form: The solution of the system of Eq. (29) may be written conveniently in the form:

31 $$\begin{aligned} v_x^*= & \sum _{n=1}^3 v_{1n} \,M_{n } e^{-\xi _{n } y}+\sum _{n=1}^3 V_{1n} \,m_{n } e^{\xi _{n } y},\end{aligned}$$

32 $$\begin{aligned} v_y^*= & \sum _{n=1}^3 v_{2 n } \, M_{n } e^{-\xi _{n }y}+\sum _{n=1}^3 V_{2n} \,m_{n } e^{\xi _{n } y},\end{aligned}$$

33 $$\begin{aligned} \theta ^*= & \sum _{n=1}^3 v_{3 n } \, M_{n } e^{-\xi _{n }y}+\sum _{n=1}^3 V_{3n} \,m_{n } e^{\xi _{n } y},\end{aligned}$$

34 $$\begin{aligned} \sigma _{yy}^*= & \sum _{n=1}^3 v_{4n } \, M_{n } e^{-\xi _{n }y}+\sum _{n=1}^3 V_{4n} \,m_{n } e^{\xi _{n } y},\end{aligned}$$

35 $$\begin{aligned} \sigma _{xy}^*= & \sum _{n=1}^3 v_{5 n } \, M_{n } e^{-\xi _{n }y}+\sum _{n=1}^3 V_{5n} \,m_{n } e^{\xi _{n } y},\end{aligned}$$

36 $$\begin{aligned} q_y^*= & \sum _{n=1}^3 v_{6n } \, M_{n }e^{-\xi _{n }y}+\sum _{n=1}^3 V_{6n} \,m_{n } e^{\xi _{n } y}. \end{aligned}$$

where $v_{in }$ and $V_{in }, i=1,2,\ldots ,6, \, n=1,2,3$ are constants given by Mathematica 14.0. The remaining two solution functions may now be calculated as explained above.

### Boundary conditions for the homogeneous system

The loading conditions of sandwich structures may be diverse, depending on design issues and uses of the structure. Static and dynamic response of sandwich structures has been investigated in numerous papers. For the sake of concreteness, the governing equations will be solved under the following conditions:

1. Thermal boundary condition:
  37 $$\begin{aligned} \theta =f_{1}^{*}e^{i\left( k x-\omega _r t\right) +\omega _i t}, \qquad \text {on } \,\, y=\pm 2\ell \end{aligned}$$
2. Mechanical boundary conditions:
  38 $$\begin{aligned} \sigma _{yy}=0,\qquad \sigma _{xy}=0, \qquad \text {on } \,\, y=\pm 2\ell \end{aligned}$$
3. Continuity conditions:
  39 $$\begin{aligned} K^I \theta ^I= & K^{II} \theta ^{II}, \qquad \text {on } \,\, y=-\ell \end{aligned}$$
  40 $$\begin{aligned} K^I \theta ^I_{,y}= & K^{II} \theta ^{II}_{,y}, \qquad \text {on } \,\, y=-\ell \end{aligned}$$
  41 $$\begin{aligned} K^{II} \theta ^{II}= & K^{III} \theta ^{III}, \qquad \text {on } \,\, y=\ell \end{aligned}$$
  42 $$\begin{aligned} K^{II}\theta ^{II}_{,y}= & K^{III}\theta ^{III}_{,y}, \qquad \text {on } \,\, y=\ell \end{aligned}$$
  43 $$\begin{aligned} \sigma _{xy}^I= & \sigma _{xy}^{II}, \qquad \text {on } \,\, y=-\ell \end{aligned}$$
  44 $$\begin{aligned} \sigma _{yy}^I= & \sigma _{yy}^{II}, \qquad \text {on } \,\, y=-\ell \end{aligned}$$
  45 $$\begin{aligned} \sigma _{xy}= & \sigma _{xy}^{III}, \qquad \text {on } \,\, y=\ell \end{aligned}$$
  46 $$\begin{aligned} \sigma _{yy}^{II}= & \sigma _{yy}^{III}, \qquad \text {on } \,\, y=\ell \end{aligned}$$
  47 $$\begin{aligned} v_{x}^I= & v_{x}^{II}, \qquad \text {on } \,\, y=-\ell \end{aligned}$$
  48 $$\begin{aligned} v_{y}^I= & v_{y}^{II}, \qquad \text {on } \,\, y=-\ell \end{aligned}$$
  49 $$\begin{aligned} v_{x}= & v_{x}^{III}, \qquad \text {on } \,\, y=\ell \end{aligned}$$
  50 $$\begin{aligned} v_{y}^{II}= & v_{y}^{III}, \qquad \text {on } \,\, y=\ell \end{aligned}$$
  where $$\ell =\frac{L}{L_0}$$.

The conditions (39) and (41) determine the jumps in the function of temperature at the interfaces.

Applying the boundary conditions to determine the $M_{i}^{I,II,III}$ and $m_{i}^{I,II,III}$ one gets the system of equations. This system is cast in the following matricial form:

51 $$\begin{aligned} \begin{pmatrix} M_{1}^{I} \\ M_{2}^{I} \\ M_{3}^{I} \\ m_{1}^{I} \\ m_{2}^{I} \\ m_{3}^{I} \\ M_{1}^{II} \\ M_{2}^{II} \\ M_{3}^{II} \\ m_{1}^{III} \\ m_{2}^{III} \\ m_{3}^{III} \\ M_{1}^{III} \\ M_{2}^{III} \\ M_{3}^{III} \\ m_{1}^{III} \\ m_{2}^{III} \\ m_{3}^{III} \\ \end{pmatrix}=\begin{pmatrix} \Lambda _1 & \Lambda _2 & \Lambda _3 \\ \Lambda _4& \Lambda _5 & \Lambda _6\\ \Lambda _7& \Lambda _8 & \Lambda _9 \\ \end{pmatrix}^{-1}\begin{pmatrix} 0\\ 0 \\ 0\\ 0 \\ 0\\ 0 \\ 0\\ 0 \\ 0\\ 0 \\ 0\\ 0 \\ f_1^\star \\ f_1^\star \\ 0\\ 0 \\ 0\\ 0 \end{pmatrix}. \end{aligned}$$

where $\Lambda _{1}-\Lambda _{9}$ are matrices given in Appendix D.

## The non-homogeneous system

$$\begin{aligned} {\left( \begin{array}{c} D v_x^{\text {**}} \\ D v_y^{\text {**}} \\ D \theta ^{\text {**}} \\ D \sigma _{yy}^{\text {**}} \\ D \sigma _{xy}^{\text {**}} \\ D q_y^{\text {**}} \\ \end{array} \right) =\left( \begin{array}{cccccc} 0 & A_{16} & 0 & 0 & A_{17} & 0 \\ A_{18} & 0 & 0 & A_{19} & 0 & 0 \\ 0 & 0 & 0 & 0 & 0 & A_{20} \\ 0 & A_{21} & 0 & 0 & A_{16} & A_{22} \\ A_{23} & 0 & A_{24} & A_{18} & 0 & 0 \\ A_{25} & 0 & A_{26} & A_{27} & 0 & 0 \\ \end{array} \right) \left( \begin{array}{c} v_x^{\text {**}} \\ v_y^{\text {**}} \\ \theta ^{\text {**}} \\ \sigma _{yy}^{\text {**}} \\ \sigma _{xy}^{\text {**}} \\ q_y^{\text {**}} \\ \end{array} \right) +\left( \begin{array}{c} 0 \\ 0 \\ A_{31} \, \theta ^{*} {\theta ^{*}}' \\ A_{32} \, \theta ^{*} {\theta ^{*}}' \\ 0 \\ A_{33} \, {\theta ^{*}} ^2 \\ \end{array} \right) } \end{aligned}$$

or

52 $$\begin{aligned} \left( \begin{array}{c} D v_x^{\text {**}} \\ D v_y^{\text {**}} \\ D \theta ^{\text {**}} \\ D \sigma _{yy}^{\text {**}} \\ D \sigma _{xy}^{\text {**}} \\ D q_y^{\text {**}} \\ \end{array} \right)= & \left( \begin{array}{cccccc} 0 & A_{16} & 0 & 0 & A_{17} & 0 \\ A_{18} & 0 & 0 & A_{19} & 0 & 0 \\ 0 & 0 & 0 & 0 & 0 & A_{20} \\ 0 & A_{21} & 0 & 0 & A_{16} & A_{22} \\ A_{23} & 0 & A_{24} & A_{18} & 0 & 0 \\ A_{25} & 0 & A_{26} & A_{27} & 0 & 0 \\ \end{array} \right) \left( \begin{array}{c} v_x^{\text {**}} \\ v_y^{\text {**}} \\ \theta ^{\text {**}} \\ \sigma _{yy}^{\text {**}} \\ \sigma _{xy}^{\text {**}} \\ q_y^{\text {**}} \\ \end{array} \right) \nonumber \\ & + \left( \begin{array}{c} 0 \\ 0 \\ \displaystyle \sum _{i=1}^3 \sum _{j=1}^3 A_{31} ( - \xi _{i } v_{3 i } v_{3 j } M_{i } M_{j } \, e^{-y \left( \xi _{i }+\xi _{j }\right) } \\ +(-\xi _{i }+\xi _{j }) v_{3 i } V_{3 j } M_{i } m_{j } \, e^{y \left( -\xi _{i }+\xi _{j }\right) } \\ +\xi _{i } V_{3 i } V_{3 j } m_{i } m_{j } \, e^{y \left( \xi _{i }+\xi _{j }\right) } ) \\ \displaystyle \sum _{i=1}^3 \sum _{j=1}^3 A_{32} (v_{3 i } v_{3 j } M_{i } M_{j } \, e^{-y \left( \xi _{i }+\xi _{j }\right) } \\ +( 2 v_{3 i } V_{3 j } M_{i } m_{j } \, e^{y \left( -\xi _{i }+\xi _{j }\right) } \\ V_{3 i } V_{3 j } m_{i } m_{j } \, e^{y \left( \xi _{i }+\xi _{j }\right) } ) \\ 0 \\ \displaystyle \sum _{i=1}^3 \sum _{j=1}^3 A_{33} ( - \xi _{i } v_{3 i } v_{3 j } M_{i } M_{j } \, e^{-y \left( \xi _{i }+\xi _{j }\right) } \\ +(-\xi _{i }+\xi _{j }) v_{3 i } V_{3 j } M_{i } m_{j } \, e^{y \left( -\xi _{i }+\xi _{j }\right) } \\ +\xi _{i } V_{3 i } V_{3 j } m_{i } m_{j } \, e^{y \left( \xi _{i }+\xi _{j }\right) } ) \\ \end{array} \right) \end{aligned}$$

Solving the homogenuous part of Eq.(52), one writes down the characteristic polynomial as:

53 $$\begin{aligned} \zeta ^6- B_1 \zeta ^4+B_2 \zeta ^2-B_3=0. \end{aligned}$$

where $B_1, B_2$ and $B_3$ are constants given by Mathematica 14.0.

Now solve for the non-homogeneous part by the method of undetermined coefficients. The particular solution is taken in the form:

$$\begin{aligned} & \sum _{i=1}^3 \sum _{j=1}^3( Q_{n,ij} v_{3 i} v_{3 j} \, M_{i } M_{j } \, e^{-y \left( \xi _{i }+\xi _{j}\right) }\\ & \quad +W_{n,ij} v_{3 i } V_{3 j } M_{i } m_{j } \, e^{y \left( -\xi _{i }+\xi _{j }\right) } \\ & \quad +O_{n,ij} V_{3 i} V_{3 j } m_{i} m_{j } \, e^{y \left( \xi _{i }+\xi _{j }\right) }), \quad n=1,2, \cdots , 6. \end{aligned}$$

Substitution of this expression into the system yields the coefficients $Q_{n,ij}$,$W_{n,ij}$ and $O_{n,ij}$. Their expressions are listed in Appendix B.

Hence the solution of the system of Eq. (52):

$$\begin{aligned} v_x^{\text {**}}= & \sum _{i=1}^3 u_{1 i}L_{i} e^{-\zeta _{i }y}+ \sum _{i=1}^3 U_{1 i}Y_{i} e^{\zeta _{i }y}+ \sum _{i=1}^3 \sum _{j=1}^3( Q_{1,ij} v_{3 i} v_{3 j} \, M_{i } M_{j } \, e^{-y \left( \xi _{i }+\xi _{j }\right) }\\ & +W_{1,ij} v_{3 i } V_{3 j } M_{i } m_{j } \, e^{y \left( -\xi _{i }+\xi _{j }\right) } +O_{1,ij} V_{3 i } V_{3 j } m_{i } m_{j } \, e^{y \left( \xi _{i }+\xi _{j }\right) }), \\ v_y^{\text {**}}= & \sum _{i=1}^3 u_{2 i}L_{i} e^{-\zeta _{i }y}+ \sum _{i=1}^3 U_{2 i}Y_{i} e^{\zeta _{i }y}+ \sum _{i=1}^3 \sum _{j=1}^3( Q_{2,ij} v_{3 i} v_{3 j} \, M_{i } M_{j } \, e^{-y \left( \xi _{i }+\xi _{j }\right) }\\ & +W_{2,ij} v_{3 i } V_{3 j } M_{i } m_{j } \, e^{y \left( -\xi _{i }+\xi _{j }\right) } +O_{2,ij} V_{3 i } V_{3 j } m_{i } m_{j } \, e^{y \left( \xi _{i }+\xi _{j }\right) }), \\ \theta ^{\text {**}}= & \sum _{i=1}^3 u_{3 i}L_{i} e^{-\zeta _{i }y}+ \sum _{i=1}^3 U_{3 i}Y_{i} e^{\zeta _{i }y}+ \sum _{i=1}^3 \sum _{j=1}^3( Q_{3,ij} v_{3 i} v_{3 j} \, M_{i } M_{j } \, e^{-y \left( \xi _{i }+\xi _{j }\right) }\\ & +W_{3,ij} v_{3 i } V_{3 j } M_{i } m_{j } \, e^{y \left( -\xi _{i }+\xi _{j }\right) } +O_{3,ij} V_{3 i} V_{3 j } m_{i } m_{j } \, e^{y \left( \xi _{i }+\xi _{j }\right) }), \\ \sigma _{yy}^{\text {**}}= & \sum _{i=1}^3 u_{4 i}L_{i} e^{-\zeta _{i }y}+ \sum _{i=1}^3 U_{4 i}Y_{i} e^{\zeta _{i }y}+ \sum _{i=1}^3 \sum _{j=1}^3( Q_{4,ij} v_{3 i} v_{3 j} \, M_{i } M_{j } \, e^{-y \left( \xi _{i }+\xi _{j }\right) }\\ & +W_{4,ij} v_{3 i } V_{3 j } M_{i } m_{j} \, e^{y \left( -\xi _{i}+\xi _{j}\right) } +O_{4,ij} V_{3 i } V_{3 j } m_{i} m_{j} \, e^{y \left( \xi _{i}+\xi _{j}\right) }), \\ \sigma _{xy}^{\text {**}}= & \sum _{i=1}^3 u_{5 i}L_{i} e^{-\zeta _{i}y}+ \sum _{i=1}^3 U_{5 i}Y_{i} e^{\zeta _{i}y}+ \sum _{i=1}^3 \sum _{j=1}^3( Q_{5,ij} v_{3 i} v_{3 j} \, M_{i } M_{j } \, e^{-y \left( \xi _{i }+\xi _{j }\right) }\\ & +W_{5,ij} v_{3 i } V_{3 j } M_{i} m_{j} \, e^{y \left( -\xi _{i}+\xi _{j}\right) } +O_{5,ij} V_{3 i} V_{3 j} m_{i } m_{j } \, e^{y \left( \xi _{i}+\xi _{j}\right) }), \\ q _{y}^{\text {**}}= & \sum _{i=1}^3 u_{6 i}L_{i} e^{-\zeta _{i}y}+ \sum _{i=1}^3 U_{6 i}Y_{i} e^{\zeta _{i}y}+ \sum _{i=1}^3 \sum _{j=1}^3( Q_{6,ij} v_{3 i} v_{3 j} \, M_{i } M_{j } \, e^{-y \left( \xi _{i }+\xi _{j }\right) }\\ & +W_{6,ij} v_{3 i } V_{3 j } M_{i } m_{j } \, e^{y \left( -\xi _{i }+\xi _{j }\right) } +O_{6,ij} V_{3 i } V_{3 j } m_{i } m_{j } \, e^{y \left( \xi _{i}+\xi _{j}\right) }), \end{aligned}$$

where $U_{nj}$ and $u_{nj}$, $n=1,2,\ldots ,6, \, j=1,2,3$ are constants given by Mathematica 14.0.

### Boundary conditions for the non-homogeneous system

Here we have taken vanishing boundary conditions, the solution at this order of approximation being generated solely by the non-homogeneous term originating from the previous order of approximation. This term will certainly introduce additional discontinuities at the interfaces, which were not present at the first order of approximation.

1. Thermal boundary condition:
  54 $$\begin{aligned} \theta =0, \qquad \text {on} \,\, y=\pm 2l \end{aligned}$$
2. Mechanical boundary conditions:
  55 $$\begin{aligned} \sigma _{yy}=0,\qquad \sigma _{xy}=0, \qquad \text {on} \,\, y=\pm 2l \end{aligned}$$
3. Continuity conditions:
  56 $$\begin{aligned} K^I \theta ^I= & K^{II} \theta ^{II}, \qquad \text {on} \,\, y=-l \end{aligned}$$
  57 $$\begin{aligned} K^I \theta ^I_{,y}= & K^{II} \theta ^{II}_{,y}, \qquad \text {on} \,\, y=-l \end{aligned}$$
  58 $$\begin{aligned} K^{II} \theta ^{II}= & K^{III} \theta ^{III}, \qquad \text {on} \,\, y=l \end{aligned}$$
  59 $$\begin{aligned} K^{II}\theta ^{II}_{,y}= & K^{III}\theta ^{III}_{,y}, \qquad \text {on} \,\, y=l \end{aligned}$$
  60 $$\begin{aligned} \sigma _{xy}^I= & \sigma _{xy}^{II}, \qquad \text {on} \,\, y=-l \end{aligned}$$
  61 $$\begin{aligned} \sigma _{yy}^I= & \sigma _{yy}^{II}, \qquad \text {on} \,\, y=-l \end{aligned}$$
  62 $$\begin{aligned} \sigma _{xy}^{II}= & \sigma _{xy}^{III}, \qquad \text {on} \,\, y=l \end{aligned}$$
  63 $$\begin{aligned} \sigma _{yy}^{II}= & \sigma _{yy}^{III}, \qquad \text {on} \,\, y=l \end{aligned}$$
  64 $$\begin{aligned} V_{x}^I= & V_{x}^{II}, \qquad \text {on} \,\, y=-l \end{aligned}$$
  65 $$\begin{aligned} V_{y}^I= & V_{y}^{II}, \qquad \text {on} \,\, y=-l \end{aligned}$$
  66 $$\begin{aligned} V_{x}^{II}= & V_{x}^{III}, \qquad \text {on} \,\, y=l \end{aligned}$$
  67 $$\begin{aligned} V_{y}^{II}= & V_{y}^{III}, \qquad \text {on} \,\, y=l. \end{aligned}$$

Applying the boundary conditions to determine the $L_{i}^{I,II,III}$ and $Y_{i}^{I,II,III}$, one gets the system of equations. This system is cast in the following matricial form:

68 $$\begin{aligned} \begin{pmatrix} L_{ 1}^{I} \\ L_{ 2}^{I} \\ L_{ 3}^{I} \\ Y_{ 1}^{I} \\ Y_{ 2}^{I} \\ Y_{ 3} ^{I} \\ L_{ 1}^{II} \\ L_{ 2}^{II} \\ L_{ 3}^{II} \\ Y_{ 1}^{II} \\ Y_{ 2}^{II} \\ Y_{ 3} ^{II} \\ L_{ 1}^{III} \\ L_{ 2}^{III} \\ L_{ 3}^{III} \\ Y_{ 1}^{III} \\ Y_{ 2}^{III} \\ Y_{ 3} ^{III} \\ \end{pmatrix}=\begin{pmatrix} \Lambda _{10} & \Lambda _{11} & \Lambda _{12} \\ \Lambda _{13}& \Lambda _{14} & \Lambda _{15}\\ \Lambda _{16}& \Lambda _{17} & \Lambda _{18} \\ \end{pmatrix}^{-1}\begin{pmatrix} g_{1}\\ g_2\\ g_3\\ g_4\\ g_5 \\ g_6 \\ g_{7}\\ g_8\\ g_9\\ g_{10}\\ g_{11} \\ g_{12} \\ g_{13}\\ g_{14}\\ g_{15} \\ g_{16} \\ g_{17}\\ g_{18}\\ \end{pmatrix} \end{aligned}$$

where $\Lambda _{10}-\Lambda _{18}$ are matrices listed in Appendix D and $g_1-g_{18}$ in Appendix C.

## Numerical results and discussion

This section is devoted to the analysis of a concrete numerical example. The following values were taken for the calculations:

$$\begin{aligned} \eta =1, \qquad f^*_1=1. \end{aligned}$$

The materials chosen for the purpose of numerical calculations are Cadmium Selenide (CdSe) for the layers I and III, and (PZT-5A) for layer II, both exhibiting hexagonal symmetry (6 mm class). The corresponding values of the material parameters are shown in Tables 1 and 2 (C.f.^26^).

**Table 1:** Values of the geometrical and the material parameters for layers I and III.

$\Theta _0=298 \, K$	$K_0=9 \, W m^{-1}.K^{-1}$	
$\rho =5504 \, kg.m^{-3}$,	$C_e =260 \, J.kg^{-1}.K^{-1}$	$\gamma =0.5 \times 10^5 \, m^3.kg^{-1}$
$\lambda =2.6 \times 10^{10} \, kg m^{-1}.s^{-2}$	$\mu =1.6 \times 10^{10} \, kg m^{-1}.s^{-2}$	
$\tau _0=1.000 \times 10^{-11} s$	$\tau _{\theta }=0.500 \times 10^{-11 }s$	$\tau _q=0.600 \times 10^{-11}s$
$k=0.2 \, \text {(wave number)}$	$\omega _r=0.5$	$\omega _i=0.5$

**Table 2:** Values of the geometrical and the material parameters for layer II.

$\Theta _0=298 \, K$	$K_s=1.5 \, W m^{-1}.K^{-1}$	
$\rho _s =7750 \, kg.m^{-3}$,	$C_e =260 \, J.kg^{-1}.K^{-1}$	$\gamma _s =0.2 \times 10^5 \, m^3.kg^{-1}$
$\lambda _s=0.8 \times 10^{10} \, kg m^{-1}.s^{-2}$	$\mu _s =0.6 \times 10^{10} \, kg m^{-1}.s^{-2}$	
$\tau _0=1.000 \times 10^{-11} s$	$\tau _{\theta }=0.500 \times 10^{-11 }s$	$\tau _q=0.600 \times 10^{-11}s$
$k=0.2 \, \text {(wave number)}$	$\omega _r=0.5$	$\omega _i=0.5$

The eight basic unknown functions and the two components of the mechanical displacement are illustrated in 2D and 3D plots. Another set of plots shows a comparison between the distributions of each of the unknown functions for three values of parameter $\eta$.

**Fig. 2:**
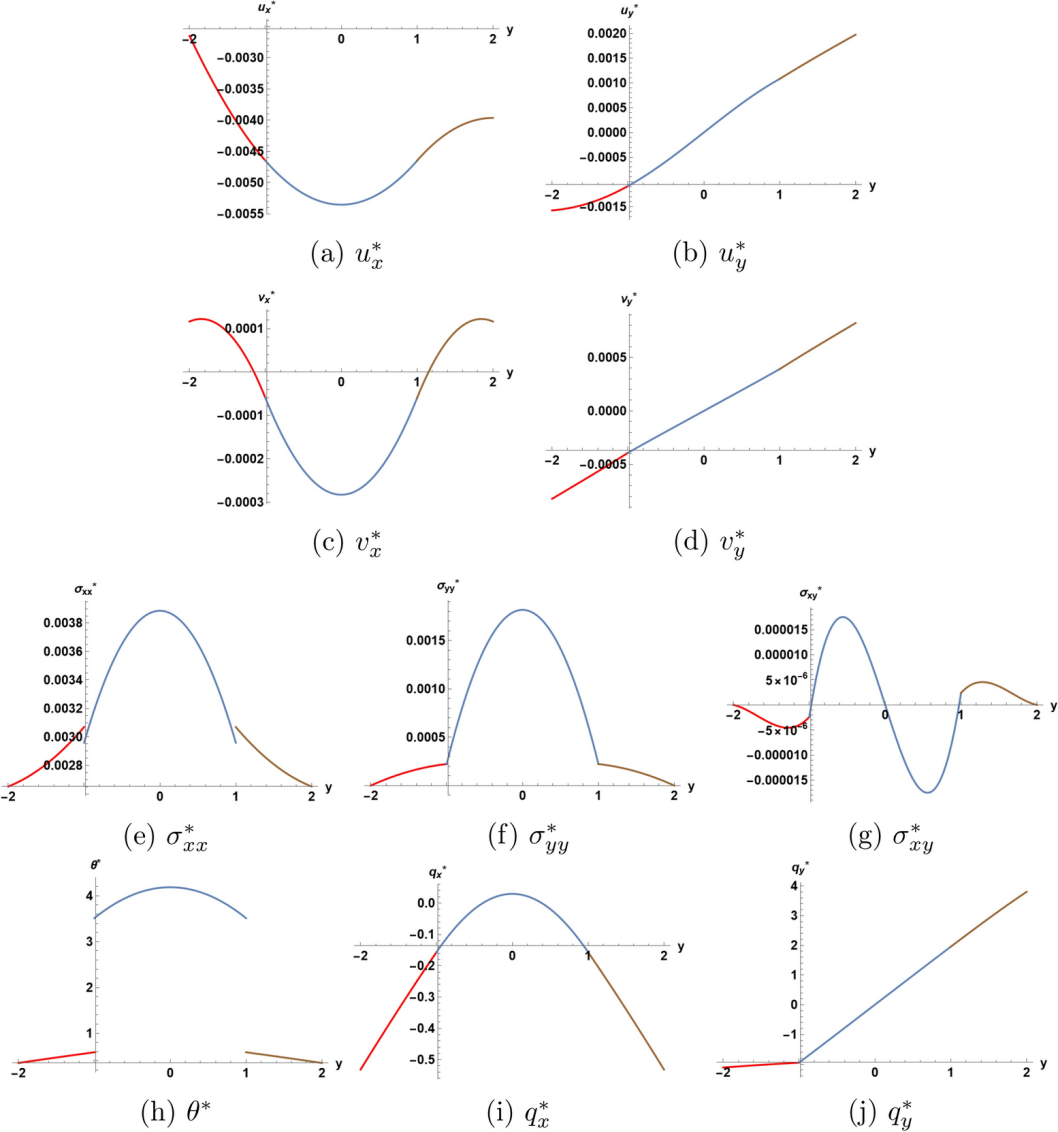
First order solution as function of *y* for $\eta = 1.0$ at $x=0.1$ and $t=1.5$.  I  II  III.

Figure 2 illustrates the first-order solution as function of the depth coordinate *y* of the structure for $\eta =1.0$, at a particular location $x=0.1$ and at a particular time moment $t=1.5$. The velocity components are continuous across the interfaces as required by the boundary conditions, the same is true for the displacement components. Only two of the eight basic unknown functions have jumps at the interfaces: the temperature $\theta ^{*}$ and the in-plane stress component $\sigma _{xx} ^{*}$ acting along the direction of wave propagation. For the particular chosen values of the location *x* and the time *t*, the structure is dilatating as shown in plots (*b*) and (*d*) for the in-depth displacement and velocity components, while the core layer has higher temperature than the two external layers as illustrated in plot (*h*). It appears from plots (*e*)-(*g*) that the in-plane stress components are higher in magnitude than the normal stress component. Like temperature, the stress component $\sigma _{xx} ^{*}$ takes its highest values in magnitude in the core layer. The stress component $\sigma _{xy} ^{*}$ in plot (*g*) is represented by a skew-symmetric curve w.r. to the center of the core layer. Except for discontinuities at the interfaces, the normal stress component $\sigma _{yy} ^{*}$ seems to have a similar behaviour like $\sigma _{xx} ^{*}$.

**Fig. 3:**
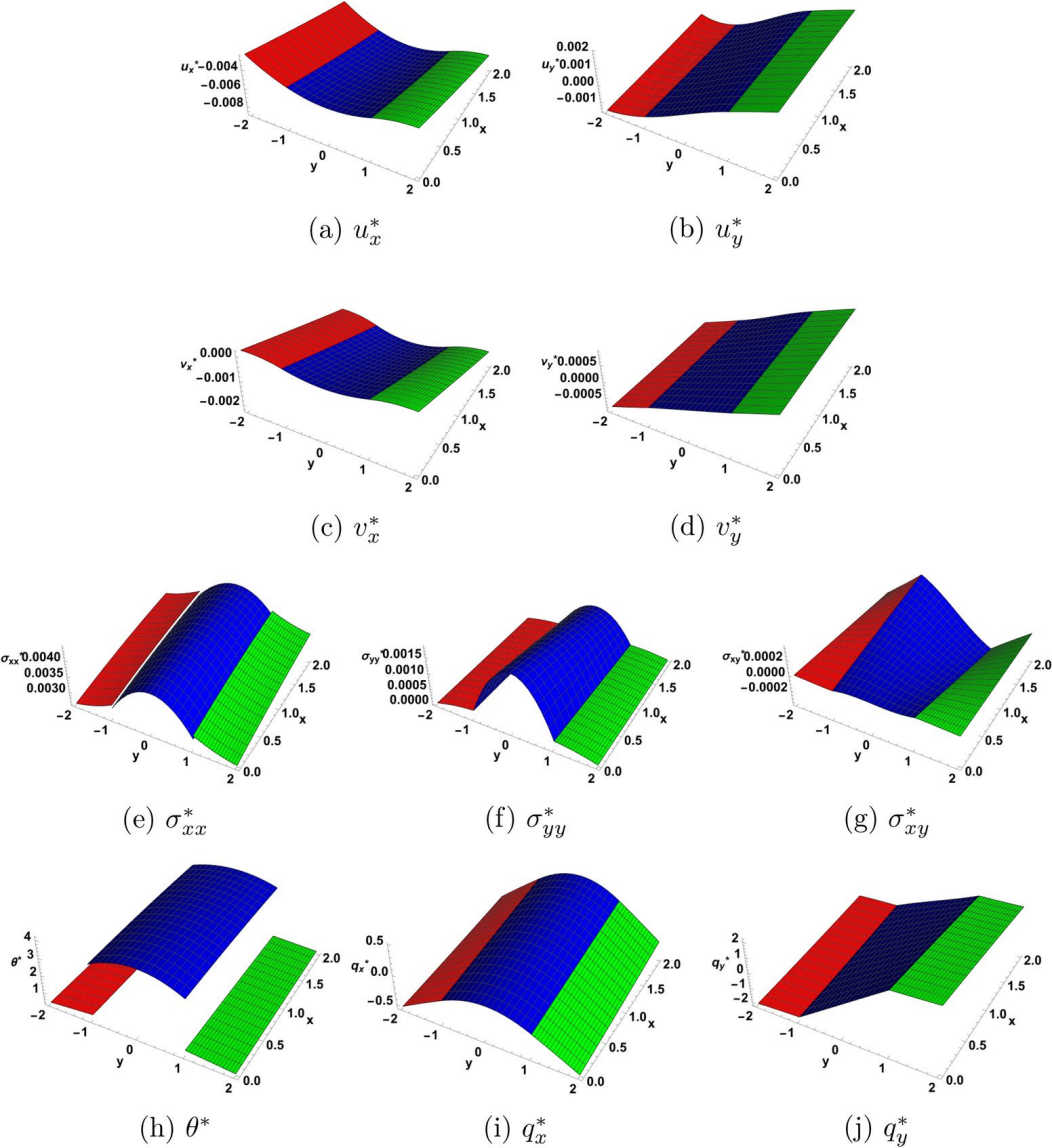
First order solution as function of (*x*, *y*) for $\eta = 1.0$ at $t=1.5$.  I  II  III.

Figure 3 is a 3D representation of the solution as function of (*x*, *y*) for $\eta =1.0$ and for a particular time moment $t=1.5,$ in which one can view the discontinuities occuring at the interfaces in the functions $\theta ^{*}$ and $\sigma _{xx} ^{*}.$ The results from^10^, which considers a three-layer sandwich structure of thermoelastic materials in extended thermodynamics seem to be qualitatively different from ours. In particular, the plot for temperature in the cited reference does not appear to have discontinuities at the interfaces. This discrepancy arises from the fact that the results in^10^ are based on the use of the continuity of heat flux as thermal boundary condition at the interfaces. This is different from the present approach, where the thermall boundary conditions at the interfaces are derived from the differential equations satisfied by the temperature (the equation of energy (5)) and by heat flux components (the Cattaneo–Vernotte equations (6) and (7)), together with the use of a boundary limiting process to derive a condition at the interface from a differential field equation, as is usually done in Continuum Mechanics.

**Fig. 4:**
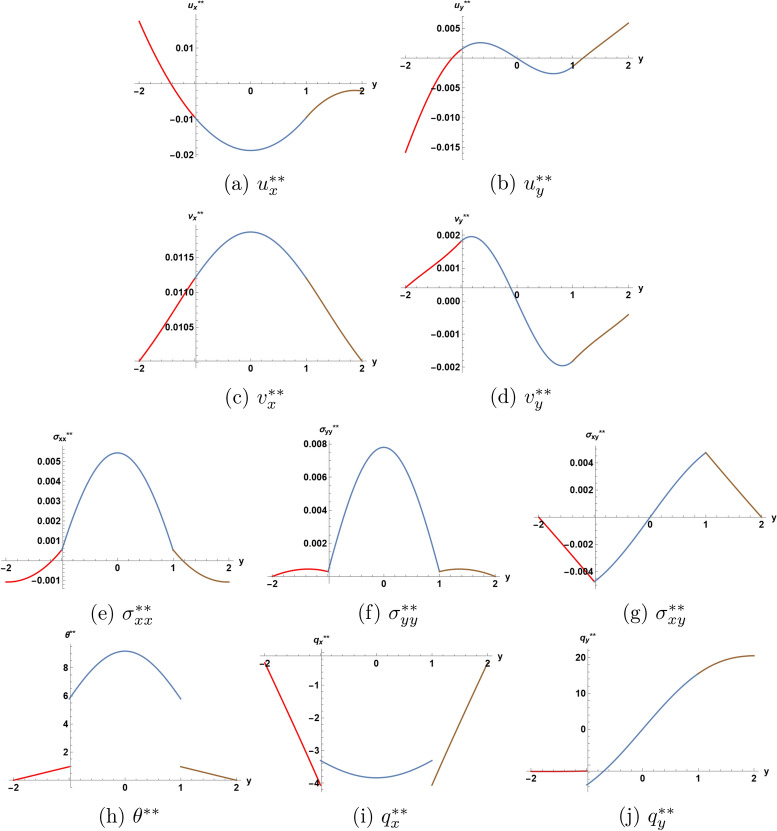
Second order solution as function of *y* for $\eta = 1.0$ at $x=0.1$ and $t=1.5$.  I  II  III.

**Fig. 5:**
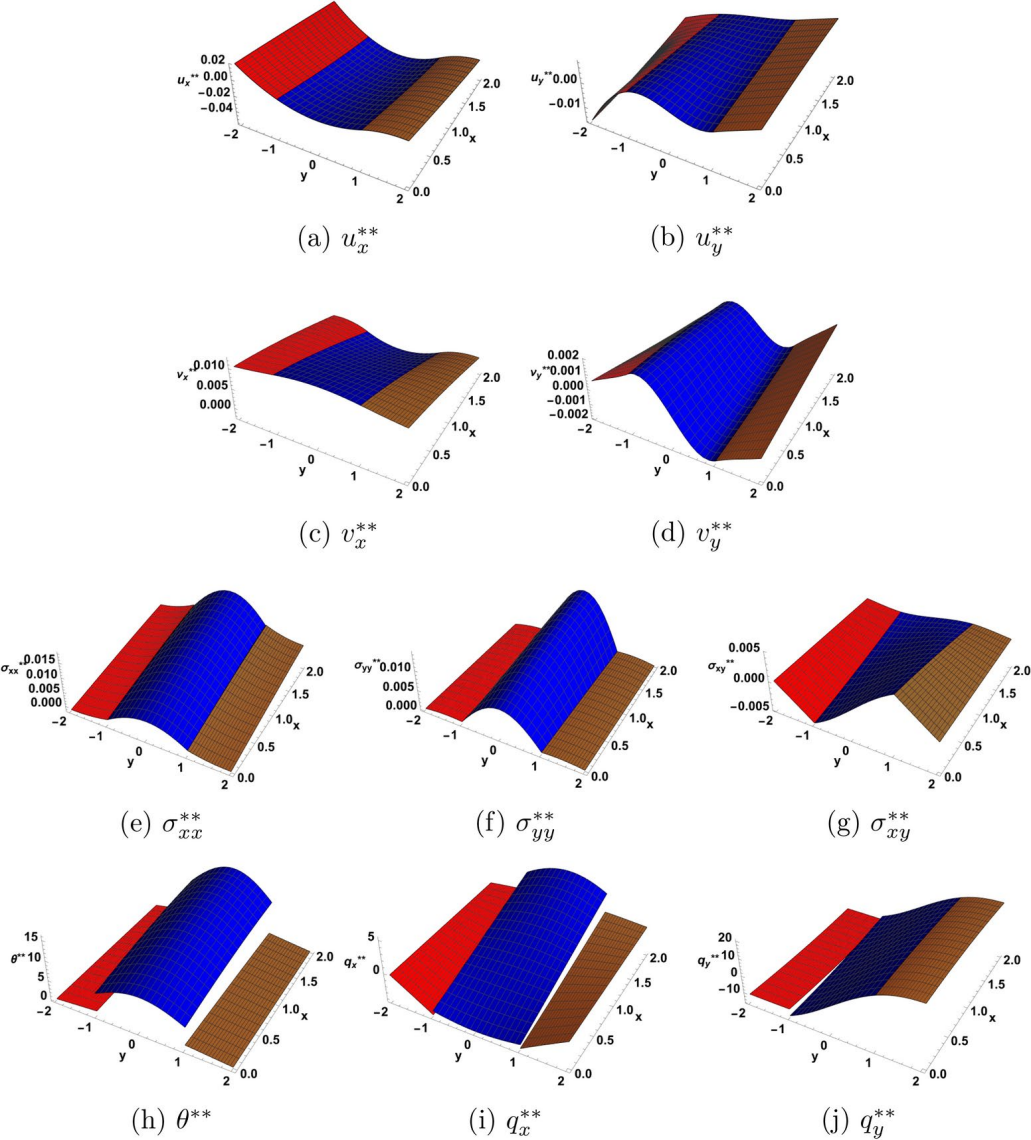
Second order solution as function of (*x*, *y*) for $\eta = 1.0$ at $t=1.5$.  I  II  III.

Figures 4 and 5 illustrate the solution at the second order of approximation. In Fig. 4, one notices that the heat flux components, which appeared to be continuous at the interfaces at the first order of approximation, now acquire jumps at the interfaces at the second order of approximation. Figure 5 provides a 3D picture of the solution, among which there are now four functions having jumps at the interfaces. This fact may be useful in determining some material constants appearing in the expressions for the solution at the second order of approximation. The measurements, however, must take in consideration the smallness of the measured quantities.

**Fig. 6:**
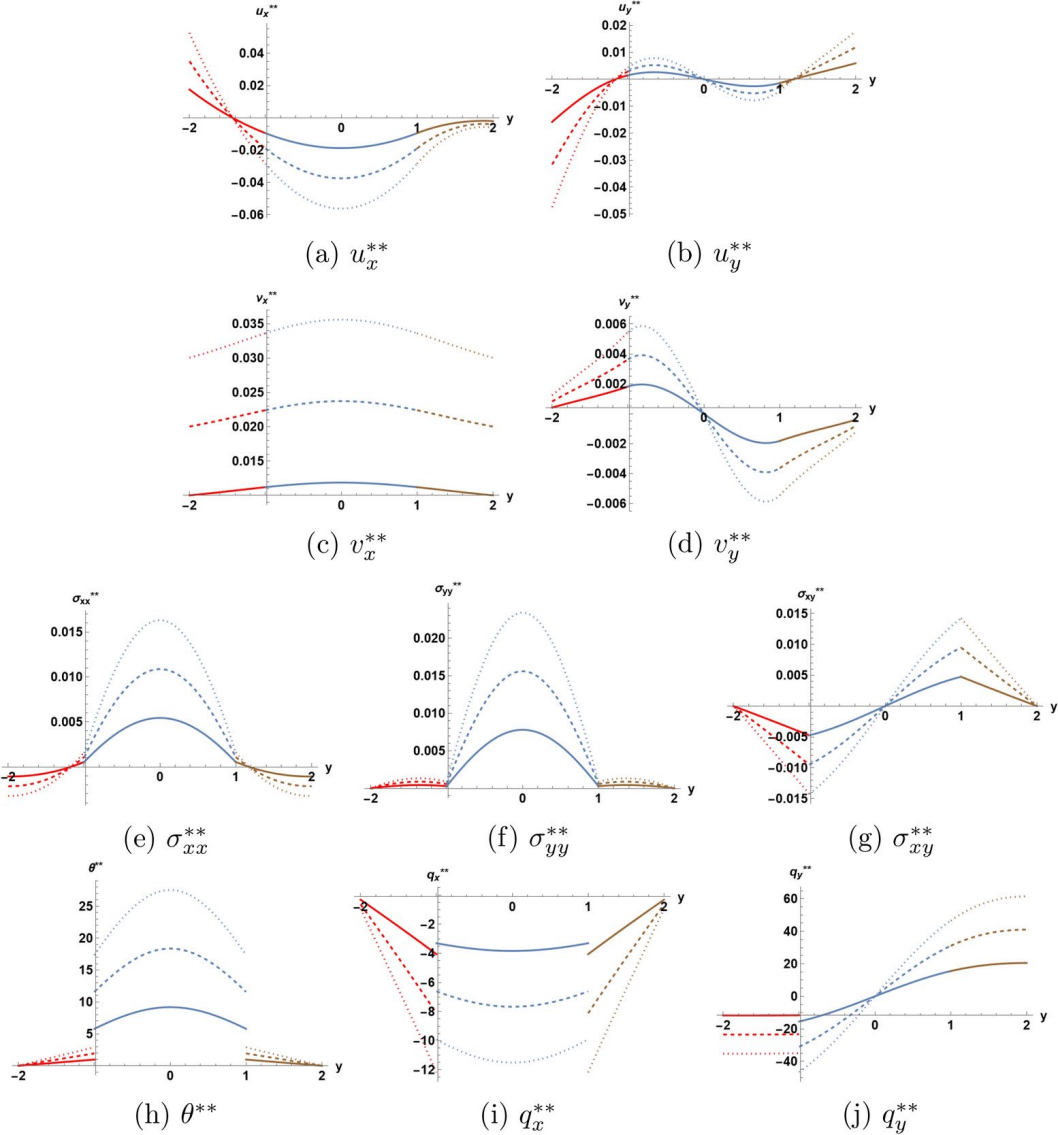
Second order solution as function of *y* at $x=0.1$ and $t=1.5$ for three values of $\eta$.  I  II  III.

Figure 6 provides a comparison of the behavior of each unknown function in the second order solution for three values of parameter $\eta$, ranging from 1.0 to 3.0. This allows to assess the effect of temperature dependence of the thermal conductivity on the appearing discontinuities at the interfaces. At a first glance, one notices tangible effects of parameter $\eta$ on the behaviour of the solution at the second order of approximation, the range of variation of the different functions seems to increase with the increase of parameter $\eta$.

The displacement and the velocity components illustrated in plots (*a*)-(*d*) show the various efects of $$\eta$$, for example relatively larger variations of the displacement component $$u_{y} ^{**}$$ towards the lower boundary of the structure, while the velocity component $$v_{x} ^{**}$$ acquires a translational increase over all the structure. The plot (*b*) for the normal displacement suggests a larger dilatation of the structure for higher values of $$\eta$$, an expected fact.

The function of temperature has jumps at the interfaces. The absolute value of these jumps increase, while the temperature increases drastically everywhere in the core layer with the increase of $\eta$. The rate of increase at the center of the core layer reaches $300 \%$ as $\eta$ varies from the value 1.0 to the value 3.0.

Both the in-plane stress component $$\sigma _{xx} ^{**}$$ and the normal stress component $$\sigma _{yy} ^{**}$$ increase everywhere in the core layer as $$\eta$$ increases. The same takes place for the stress component $$\sigma _{xy} ^{**}$$ in magnitude at the interfaces, but the values of this stress component change very little at the center of the core layer as $$\eta$$ varies from the value 1.0 to the value 3.0.

Finally, the plots (*i*) and (*j*) indicate that the effect of the variation of $\eta$ is to increase the magnitudes of the jumps at the interfaces.

If the present results are to used for an experimental determination of coefficxient $\eta$, the only efficient way is to take in consideration the jumps occuring in the heat flux components at the interfaces, since these jumps appear only at the second order of approximation.

## Conclusions

Previous work in^25,26^ has been extended to investigate nonlinear Rayleigh wave propagation in three-layer sandwich structures of thermoelastic materials with temperature dependent thermal conductivities, within dual-phase-lag. The thermal conductivity is taken to have the same linear dependence on temperature in the three layers. A particular wave solution for eight unknown functions is obtained in the form of Poincaré small parameter expansions with definite frequency and damping parameter. Only the first two orders of approximation are considered. This solution is presented and plots in 2D and 3D are provided and discussed. As a result of application of general methods of Continuum Mechanics, the temperature and the in-plane stress component along the direction of wave propagation acquire jumps at both interfaces at the first order of approximation. All other unknown functions are continuous at the interfaces, but may have there discontinuous first derivatives w.r.t. the depth coordinate. At the second order approximation, both heat flux components develop jumps at the interfaces. This behaviour at the interfaces is in contrast to that of other work, where the solution obtained under continuity of the heat flux at the interfaces. The appearance of jumps in the heat flux at the interfaces may be favourably used to carry out measurements for the determination of the material parameter $\eta$.

## Supplementary Information

Supplementary Information.

## Data Availability

All data generated or analyzed during this study are included in this article.
