# Supplementary material for: Nonlinear Rayleigh wave propagation in a three-layer sandwich structure in dual-phase-lag
Source: Sci Rep. 2024 Nov 7;14:27134. doi: 10.1038/s41598-024-73912-9 (PMC11543657; doi:10.1038/s41598-024-73912-9)
Supplement: Supplementary file 1 — Supplementary Information. [file 41598_2024_73912_MOESM1_ESM.pdf]

Appendix A

Coefficients 1

$$A_1 = -ik, \qquad A_2 = -\omega_r - i\omega_i, \qquad A_3 = \frac{-ik\alpha}{\beta}, \qquad A_4 = \frac{(\alpha-\beta)(\alpha+\beta)(\omega_r+i\omega_i)}{\beta},$$

$$A_5 = \frac{1-(\omega_r+i\omega_i)\tau_q}{K_0(-1+(\omega_r+i\omega_i)\tau_\theta)}, \qquad A_6 = -\frac{(\omega_r+i\omega_i)}{\beta_1}, \qquad A_7 = -\frac{\beta_4(-1+(\omega_r+i\omega_i)\tau_q)}{K_0(-1+(\omega_r+i\omega_i)\tau_\theta)},$$

$$A_8 = -\frac{k^2}{\beta(\omega_r+i\omega_i)} - \frac{(\omega_r+i\omega_i)}{\beta_1}, \qquad A_9 = ik\beta_4, \qquad A_{11} = \left((\omega_r + i\omega_i) - \frac{k^2K_0(-1+(\omega_r+i\omega_i)\tau_\theta)}{-1+(\omega_r+i\omega_i)\tau_q}\right),$$

$$A_{10} = \frac{(\alpha-\beta)\beta_2(ik)}{\beta}, \qquad A_{12} = \frac{(-\alpha+\beta)(\alpha+\beta)(\omega_r+i\omega_i)\beta_2}{\beta}, \qquad A_{13} = \frac{k}{\beta(i\omega_r-\omega_i)}, \qquad A_{14} = \frac{\alpha}{\beta},$$

$$A_{15} = \frac{kK_0(i+(-i\omega_r+\omega_i)\tau_\theta)}{-1+(\omega_r+i\omega_i)\tau_q}, \qquad A_{16} = -i2k, \qquad A_{17} = -2\left(\omega_r + i\omega_i\right), A_{18} = -\frac{i2\ k\alpha}{\beta},$$

$$A_{19} = \left(-2\beta\left(\omega_r + i\omega_i\right)\right) + \frac{\alpha(2\alpha(\omega_r+i\omega_i))}{\beta}, \qquad A_{20} = \frac{(1-2(\omega_r+i\omega_i)\tau_q)}{K_0(-1+2(\omega_r+i\omega_i)\tau_\theta)},$$

$$A_{21} = -\frac{2(\omega_r+i\omega_i)}{\beta_1}, \qquad A_{22} = \frac{\beta_4(1-2(\omega_r+i\omega_i)\tau_q)}{K_0(-1+2(\omega_r+i\omega_i)\tau_\theta)}, \qquad A_{23} = -\frac{2k^2}{\beta(\omega_r+i\omega_i)} - \frac{2(\omega_r+i\omega_i)}{\beta_1},$$

$$A_{24} = 2ik\beta_4, \qquad A_{25} = \frac{2ik(\alpha-\beta)\beta_2}{\beta}, \qquad A_{26} = 2\left(\omega_r + i\omega_i\right) - \frac{4k^2K_0(-1+2(\omega_r+i\omega_i)\tau_\theta)}{-1+2(\omega_r+i\omega_i)\tau_q},$$

$$A_{27} = -\frac{2\beta_2(\alpha-\beta)(\alpha+\beta)(\omega_r+i\omega_i)}{\beta}, \qquad A_{28} = -\frac{ik}{\beta(\omega_r+i\omega_i)}, \qquad A_{29} = \frac{\alpha}{\beta},$$

$$A_{30} = \frac{2kK_0(1+\tau_\theta(-2i\omega_i-2\omega_r))}{i+2\tau_q(\omega_i-i\omega_r)}, \qquad A_{31} = \eta\frac{1}{2}\left(-1 + \frac{1}{-1+2\tau_\theta(i\omega_i+\omega_r)}\right),$$

$$A_{32} = \eta\frac{1}{2}\beta_4\left(-1 + \frac{1}{-1+2\tau_\theta(i\omega_i+\omega_r)}\right), \qquad A_{33} = -\eta\frac{2k^2K_0(-1+\tau_\theta(i\omega_i+\omega_r))}{-1+2\tau_q(i\omega_i+\omega_r)}, \qquad A_{34} = \eta\frac{kK_0(1+\tau_\theta(-i\omega_i-\omega_r))}{i+2\tau_q(\omega_i-i\omega_r)}.$$

Appendix B

Coefficients 2

$$\begin{pmatrix} Q_{1,ij} \\ Q_{2,ij} \\ Q_{3,ij} \\ Q_{4,ij} \\ Q_{5,ij} \\ Q_{6,ij} \end{pmatrix} = \begin{pmatrix} \xi_{is}+\xi_{js} & A_{16} & 0 & 0 & A_{17} & 0 \\ A_{18} & \xi_{is}+\xi_{js} & 0 & A_{19} & 0 & 0 \\ 0 & 0 & \xi_{is}+\xi_{js} & 0 & 0 & A_{20} \\ 0 & A_{21} & 0 & \xi_{is}+\xi_{js} & A_{16} & A_{22} \\ A_{23} & 0 & A_{24} & A_{18} & \xi_{is}+\xi_{js} & 0 \\ A_{25} & 0 & A_{26} & A_{27} & 0 & \xi_{is}+\xi_{js} \end{pmatrix}^{-1} \begin{pmatrix} 0 \\ 0 \\ A_{31}\xi_{is} \\ A_{32}\xi_{is} \\ 0 \\ -A_{33} \end{pmatrix},$$

$$\begin{pmatrix} W_{1,ij} \\ W_{2,ij} \\ W_{3,ij} \\ W_{4,ij} \\ W_{5,ij} \\ W_{6,ij} \end{pmatrix} = \begin{pmatrix} \xi_{is}-\xi_{js} & A_{16} & 0 & 0 & A_{17} & 0 \\ A_{18} & \xi_{is}-\xi_{js} & 0 & A_{19} & 0 & 0 \\ 0 & 0 & \xi_{is}-\xi_{js} & 0 & 0 & A_{20} \\ 0 & A_{21} & 0 & \xi_{is}-\xi_{js} & A_{16} & A_{22} \\ A_{23} & 0 & A_{24} & A_{18} & \xi_{is}-\xi_{js} & 0 \\ A_{25} & 0 & A_{26} & A_{27} & 0 & \xi_{is}-\xi_{js} \end{pmatrix}^{-1} \begin{pmatrix} 0 \\ 0 \\ A_{31}(\xi_{is}-\xi_{js}) \\ A_{32}(\xi_{is}-\xi_{js}) \\ 0 \\ -2A_{33} \end{pmatrix},$$

$$\begin{pmatrix} O_{1,ij} \\ O_{2,ij} \\ O_{3,ij} \\ O_{4,ij} \\ O_{5,ij} \\ O_{6,ij} \end{pmatrix} = \begin{pmatrix} -(\xi_{is}+\xi_{js}) & A_{16} & 0 & 0 & A_{17} & 0 \\ A_{18} & -(\xi_{is}+\xi_{js}) & 0 & A_{19} & 0 & 0 \\ 0 & 0 & -(\xi_{is}+\xi_{js}) & 0 & 0 & A_{20} \\ 0 & A_{21} & 0 & -(\xi_{is}+\xi_{js}) & A_{16} & A_{22} \\ A_{23} & 0 & A_{24} & A_{18} & -(\xi_{is}+\xi_{js}) & 0 \\ A_{25} & 0 & A_{26} & A_{27} & 0 & -(\xi_{is}+\xi_{js}) \end{pmatrix}^{-1} \begin{pmatrix} 0 \\ 0 \\ -A_{31}\xi_{is} \\ -A_{32}\xi_{is} \\ 0 \\ -A_{33} \end{pmatrix}.$$

Appendix C

Coefficients 3

$$
\begin{aligned}
g_1 = &\sum_{i=1}^{3}\sum_{j=1}^{3}(Q_{1,ij}v_{3i}v_{3j}\, M_iM_j\, e^{\ell(-\xi_i-\xi_j)} + W_{1,ij}v_{3i}V_{3j}M_im_j\, e^{-\ell(-\xi_i+\xi_j)} + O_{1,ij}V_{3i}V_{3j}m_im_j\, e^{\ell(\xi_i+\xi_j)})^{I} \\
- &\sum_{i=1}^{3}\sum_{j=1}^{3}(Q_{1,ij}v_{3i}v_{3j}\, M_iM_j\, e^{\ell(-\xi_i-\xi_j)} + W_{1,ij}v_{3i}V_{3j}M_im_j\, e^{-\ell(-\xi_i+\xi_j)} + O_{1,ij}V_{3i}V_{3j}m_im_j\, e^{\ell(\xi_i+\xi_j)})^{II},
\end{aligned}
$$

$$
\begin{aligned}
g_2 = &\sum_{i=1}^{3}\sum_{j=1}^{3}(Q_{2,ij}v_{3i}v_{3j}\, M_iM_j\, e^{\ell(-\xi_i-\xi_j)} + W_{2,ij}v_{3i}V_{3j}M_im_j\, e^{-\ell(-\xi_i+\xi_j)} + O_{2,ij}V_{3i}V_{3j}m_im_j\, e^{\ell(\xi_i+\xi_j)})^{I} \\
- &\sum_{i=1}^{3}\sum_{j=1}^{3}(Q_{2,ij}v_{3i}v_{3j}\, M_iM_j\, e^{\ell(-\xi_i-\xi_j)} + W_{2,ij}v_{3i}V_{3j}M_im_j\, e^{-\ell(-\xi_i+\xi_j)} + O_{2,ij}V_{3i}V_{3j}m_im_j\, e^{\ell(\xi_i+\xi_j)})^{II},
\end{aligned}
$$

$$
\begin{aligned}
g_3 = &\sum_{i=1}^{3}\sum_{j=1}^{3}(Q_{1,ij}v_{3i}v_{3j}\, M_iM_j\, e^{-\ell(-\xi_i-\xi_j)} + W_{1,ij}v_{3i}V_{3j}M_im_j\, e^{\ell(-\xi_i+\xi_j)} + O_{1,ij}V_{3i}V_{3j}m_im_j\, e^{-\ell(\xi_i+\xi_j)})^{II} \\
- &\sum_{i=1}^{3}\sum_{j=1}^{3}(Q_{1,ij}v_{3i}v_{3j}\, M_iM_j\, e^{-\ell(-\xi_i-\xi_j)} + W_{1,ij}v_{3i}V_{3j}M_im_j\, e^{\ell(-\xi_i+\xi_j)} + O_{1,ij}V_{3i}V_{3j}m_im_j\, e^{-\ell(\xi_i+\xi_j)})^{III},
\end{aligned}
$$

$$
\begin{aligned}
g_4 = &\sum_{i=1}^{3}\sum_{j=1}^{3}(Q_{2,ij}v_{3i}v_{3j}\, M_iM_j\, e^{-\ell(-\xi_i-\xi_j)} + W_{2,ij}v_{3i}V_{3j}M_im_j\, e^{\ell(-\xi_i+\xi_j)} + O_{2,ij}V_{3i}V_{3j}m_im_j\, e^{-\ell(\xi_i+\xi_j)})^{II} \\
- &\sum_{i=1}^{3}\sum_{j=1}^{3}(Q_{2,ij}v_{3i}v_{3j}\, M_iM_j\, e^{-\ell(-\xi_i-\xi_j)} + W_{2,ij}v_{3i}V_{3j}M_im_j\, e^{\ell(-\xi_i+\xi_j)} + O_{2,ij}V_{3i}V_{3j}m_im_j\, e^{-\ell(\xi_i+\xi_j)})^{III},
\end{aligned}
$$

$$
\begin{aligned}
g_5 = &\sum_{i=1}^{3}\sum_{j=1}^{3}(Q_{4,ij}u_{3i}u_{3j}\, M_iM_j\, e^{\ell(-\zeta_i-\zeta_j)} + W_{4,ij}u_{3i}U_{3j}M_im_j\, e^{-\ell(-\zeta_i+\zeta_j)} + O_{4,ij}U_{3i}U_{3j}m_im_j\, e^{\ell(\zeta_i+\zeta_j)})^{I} \\
- &\sum_{i=1}^{3}\sum_{j=1}^{3}(Q_{4,ij}u_{3i}u_{3j}\, M_iM_j\, e^{\ell(-\zeta_i-\zeta_j)} + W_{4,ij}u_{3i}U_{3j}M_im_j\, e^{-\ell(-\zeta_i+\zeta_j)} + O_{4,ij}U_{3i}U_{3j}m_im_j\, e^{\ell(\zeta_i+\zeta_j)})^{II},
\end{aligned}
$$

$$
\begin{aligned}
g_6 = &\sum_{i=1}^{3}\sum_{j=1}^{3}(Q_{5,ij}u_{3i}u_{3j}\, M_iM_j\, e^{\ell(-\zeta_i-\zeta_j)} + W_{5,ij}u_{3i}U_{3j}M_im_j\, e^{-\ell(-\zeta_i+\zeta_j)} + O_{5,ij}U_{3i}U_{3j}m_im_j\, e^{\ell(\zeta_i+\zeta_j)})^{I} \\
- &\sum_{i=1}^{3}\sum_{j=1}^{3}(Q_{5,ij}u_{3i}u_{3j}\, M_iM_j\, e^{\ell(-\zeta_i-\zeta_j)} + W_{5,ij}u_{3i}U_{3j}M_im_j\, e^{-\ell(-\zeta_i+\zeta_j)} + O_{5,ij}U_{3i}U_{3j}m_im_j\, e^{\ell(\zeta_i+\zeta_j)})^{II},
\end{aligned}
$$

$$
\begin{aligned}
g_7 = &\sum_{i=1}^{3}\sum_{j=1}^{3}(Q_{4,ij}u_{3i}u_{3j}\, M_iM_j\, e^{-\ell(-\zeta_i-\zeta_j)} + W_{4,ij}u_{3i}V_{3j}M_im_j\, e^{\ell(-\zeta_i+\zeta_j)} + O_{4,ij}V_{3i}V_{3j}m_im_j\, e^{-\ell(\zeta_i+\zeta_j)})^{II} \\
- &\sum_{i=1}^{3}\sum_{j=1}^{3}(Q_{4,ij}u_{3i}u_{3j}\, M_iM_j\, e^{-\ell(-\zeta_i-\zeta_j)} + W_{4,ij}u_{3i}V_{3j}M_im_j\, e^{\ell(-\zeta_i+\zeta_j)} + O_{4,ij}V_{3i}V_{3j}m_im_j\, e^{-\ell(\zeta_i+\zeta_j)})^{III},
\end{aligned}
$$

$$
\begin{aligned}
g_8 \;=\; & \sum_{i=1}^{3}\sum_{j=1}^{3}(Q_{5,ij}u_{3i}u_{3j}\, M_iM_j\, e^{-\ell(-\zeta_i-\zeta_j)} + W_{5,ij}u_{3i}V_{3j}M_im_j\, e^{\ell(-\zeta_i+\zeta_j)} + O_{5,ij}V_{3i}V_{3j}m_im_j\, e^{-\ell(\zeta_i+\zeta_j)})^{II} \\
& - \sum_{i=1}^{3}\sum_{j=1}^{3}(Q_{5,ij}u_{3i}u_{3j}\, M_iM_j\, e^{-\ell(-\zeta_i-\zeta_j)} + W_{5,ij}u_{3i}V_{3j}M_im_j\, e^{\ell(-\zeta_i+\zeta_j)} + O_{5,ij}V_{3i}V_{3j}m_im_j\, e^{-\ell(\zeta_i+\zeta_j)})^{III},
\end{aligned}
$$

$$
\begin{aligned}
g_9 \;=\; & K^I\sum_{i=1}^{3}\sum_{j=1}^{3}(Q_{3,ij}v_{3i}v_{3j}\, M_iM_j\, e^{\ell(-\xi_i-\xi_j)} + W_{3,ij}v_{3i}V_{3j}M_im_j\, e^{-\ell(-\xi_i+\xi_j)} + O_{3,ij}V_{3i}V_{3j}m_im_j\, e^{\ell(\xi_i+\xi_j)})^{I} \\
& - K^{II}\sum_{i=1}^{3}\sum_{j=1}^{3}(Q_{3,ij}v_{3i}v_{3j}\, M_iM_j\, e^{\ell(-\xi_i-\xi_j)} + W_{3,ij}v_{3i}V_{3j}M_im_j\, e^{-\ell(-\xi_i+\xi_j)} + O_{3,ij}V_{3i}V_{3j}m_im_j\, e^{\ell(\xi_i+\xi_j)})^{II},
\end{aligned}
$$

$$
\begin{aligned}
g_{10} \;=\; & K^I\sum_{i=1}^{3}\sum_{j=1}^{3}((-\xi_i-\xi_j)\, Q_{3,ij}v_{3i}v_{3j}\, M_iM_j\, e^{\ell(-\xi_i-\xi_j)} \\
& + (-\xi_i+\xi_j)\, W_{3,ij}v_{3i}V_{3j}M_im_j\, e^{-\ell(-\xi_i+\xi_j)} + (\xi_i+\xi_j)\, O_{3,ij}V_{3i}V_{3j}m_im_j\, e^{\ell(\xi_i+\xi_j)})^{I} \\
& - K^{II}\sum_{i=1}^{3}\sum_{j=1}^{3}((-\xi_i-\xi_j)\, Q_{3,ij}v_{3i}v_{3j}\, M_iM_j\, e^{\ell(-\xi_i-\xi_j)} \\
& + (-\xi_i+\xi_j)\, W_{3,ij}v_{3i}V_{3j}M_im_j\, e^{-\ell(-\xi_i+\xi_j)} + (\xi_i+\xi_j)\, O_{3,ij}V_{3i}V_{3j}m_im_j\, e^{\ell(\xi_i+\xi_j)})^{II},
\end{aligned}
$$

$$
\begin{aligned}
g_{11} \;=\; & K^{II}\sum_{i=1}^{3}\sum_{j=1}^{3}(Q_{3,ij}v_{3i}v_{3j}\, M_iM_j\, e^{\ell(-\xi_i-\xi_j)} + W_{3,ij}v_{3i}V_{3j}M_im_j\, e^{-\ell(-\xi_i+\xi_j)} + O_{3,ij}V_{3i}V_{3j}m_im_j\, e^{\ell(\xi_i+\xi_j)})^{II} \\
& - K^{III}\sum_{i=1}^{3}\sum_{j=1}^{3}(Q_{3,ij}v_{3i}v_{3j}\, M_iM_j\, e^{\ell(-\xi_i-\xi_j)} + W_{3,ij}v_{3i}V_{3j}M_im_j\, e^{-\ell(-\xi_i+\xi_j)} + O_{3,ij}V_{3i}V_{3j}m_im_j\, e^{\ell(\xi_i+\xi_j)})^{III},
\end{aligned}
$$

$$
\begin{aligned}
g_{12} \;=\; & K^{II}\sum_{i=1}^{3}\sum_{j=1}^{3}((-\xi_i-\xi_j)\, Q_{3,ij}v_{3i}v_{3j}\, M_iM_j\, e^{\ell(-\xi_i-\xi_j)} \\
& + (-\xi_i+\xi_j)\, W_{3,ij}v_{3i}V_{3j}M_im_j\, e^{-\ell(-\xi_i+\xi_j)} + (\xi_i+\xi_j)\, O_{3,ij}V_{3i}V_{3j}m_im_j\, e^{\ell(\xi_i+\xi_j)})^{II} \\
& - K^{III}\sum_{i=1}^{3}\sum_{j=1}^{3}((-\xi_i-\xi_j)\, Q_{3,ij}v_{3i}v_{3j}\, M_iM_j\, e^{\ell(-\xi_i-\xi_j)} \\
& + (-\xi_i+\xi_j)\, W_{3,ij}v_{3i}V_{3j}M_im_j\, e^{-\ell(-\xi_i+\xi_j)} + (\xi_i+\xi_j)\, O_{3,ij}V_{3i}V_{3j}m_im_j\, e^{\ell(\xi_i+\xi_j)})^{III},
\end{aligned}
$$

$$
g_{13} = \sum_{i=1}^{3}\sum_{j=1}^{3}(Q_{3,ij}v_{3i}v_{3j}\, M_iM_j\, e^{2\ell(-\xi_i-\xi_j)} + W_{3,ij}v_{3i}V_{3j}M_im_j\, e^{-2\ell(-\xi_i+\xi_j)} + O_{3,ij}V_{3i}V_{3j}m_im_j\, e^{2\ell(\xi_i+\xi_j)})^{I},
$$

$$
g_{14} = \sum_{i=1}^{3}\sum_{j=1}^{3}(Q_{3,ij}v_{3i}v_{3j}\, M_iM_j\, e^{-2\ell(-\xi_i-\xi_j)} + W_{3,ij}v_{3i}V_{3j}M_im_j\, e^{2\ell(-\xi_i+\xi_j)} + O_{3,ij}V_{3i}V_{3j}m_im_j\, e^{-2\ell(\xi_i+\xi_j)})^{III},
$$

$$
g_{15} = \sum_{i=1}^{3}\sum_{j=1}^{3}(Q_{4,ij}v_{3i}v_{3j}\, M_iM_j\, e^{2\ell(-\xi_i-\xi_j)} + W_{4,ij}v_{3i}V_{3j}M_im_j\, e^{-2\ell(-\xi_i+\xi_j)} + O_{4,ij}V_{3i}V_{3j}m_im_j\, e^{2\ell(\xi_i+\xi_j)})^{I},
$$

$$g_{16} = \sum_{i=1}^{3}\sum_{j=1}^{3}(Q_{4,ij}v_{3i}v_{3j}\, M_i M_j\, e^{-2\ell(-\xi_i-\xi_j)} + W_{4,ij}v_{3i}V_{3j}M_i m_j\, e^{2\ell(-\xi_i+\xi_j)} + O_{4,ij}V_{3i}V_{3j}m_i m_j\, e^{-2\ell(\xi_i+\xi_j)})^{III},$$

$$g_{17} = \sum_{i=1}^{3}\sum_{j=1}^{3}(Q_{5,ij}v_{3i}v_{3j}\, M_i M_j\, e^{2\ell(-\xi_i-\xi_j)} + W_{5,ij}v_{3i}V_{3j}M_i m_j\, e^{-2\ell(-\xi_i+\xi_j)} + O_{5,ij}V_{3i}V_{3j}m_i m_j\, e^{2\ell(\xi_i+\xi_j)})^{I},$$

$$g_{18} = \sum_{i=1}^{3}\sum_{j=1}^{3}(Q_{5,ij}v_{3i}v_{3j}\, M_i M_j\, e^{-2\ell(-\xi_i-\xi_j)} + W_{5,ij}v_{3i}V_{3j}M_i m_j\, e^{2\ell(-\xi_i+\xi_j)} + O_{5,ij}V_{3i}V_{3j}m_i m_j\, e^{-2\ell(\xi_i+\xi_j)})^{III},$$

Appendix D

Coefficients 4

$$\Lambda_1 = \begin{pmatrix} v_{11}^I e^{-\xi_1^I(-\ell)} & v_{12}^I e^{-\xi_2^I(-\ell)} & v_{13}^I e^{-\xi_3^I(-\ell)} & V_{11}^I e^{\xi_1^I(-\ell)} & V_{12}^I e^{\xi_2^I(-\ell)} & V_{13}^I e^{\xi_3^I(-\ell)} \\ v_{21}^I e^{-\xi_1^I(-\ell)} & v_{22}^I e^{-\xi_2^I(-\ell)} & v_{23}^I e^{-\xi_3^I(-\ell)} & V_{21}^I e^{\xi_1^I(-\ell)} & V_{22}^I e^{\xi_2^I(-\ell)} & V_{23}^I e^{\xi_3^I(-\ell)} \\ 0 & 0 & 0 & 0 & 0 & 0 \\ 0 & 0 & 0 & 0 & 0 & 0 \\ v_{41}^I e^{-\xi_1^I(-\ell)} & v_{42}^I e^{-\xi_2^I(-\ell)} & v_{43}^I e^{-\xi_3^I(-\ell)} & V_{41}^I e^{\xi_1^I(-\ell)} & V_{42}^I e^{\xi_2^I(-\ell)} & V_{43}^I e^{\xi_3^I(-\ell)} \\ v_{51}^I e^{-\xi_1^I(-\ell)} & v_{52}^I e^{-\xi_2^I(-\ell)} & v_{53}^I e^{-\xi_3^I(-\ell)} & V_{51}^I e^{\xi_1^I(-\ell)} & V_{52}^I e^{\xi_2^I(-\ell)} & V_{53}^I e^{\xi_3^I(-\ell)} \end{pmatrix},$$

$$\Lambda_2 = \begin{pmatrix} v_{11}^{II} e^{-\xi_1^{II}(-\ell)} & v_{12}^{II} e^{-\xi_2^{II}(-\ell)} & v_{13}^{II} e^{-\xi_3^{II}(-\ell)} & V_{11}^{II} e^{\xi_1^{II}(-\ell)} & V_{12}^{II} e^{\xi_2^{II}(-\ell)} & V_{13}^{II} e^{\xi_3^{II}(-\ell)} \\ v_{21}^{II} e^{-\xi_1^{Ii}(-\ell)} & v_{22}^{II} e^{-\xi_2^{II}(-\ell)} & v_{23}^{II} e^{-\xi_3^{II}(-\ell)} & V_{21}^{II} e^{\xi_1^{II}(-\ell)} & V_{22}^{II} e^{\xi_2^{II}(-\ell)} & V_{23}^{II} e^{\xi_3^{II}(-\ell)} \\ v_{11}^{II} e^{-\xi_1^{II}(\ell)} & v_{12}^{II} e^{-\xi_2^{II}(\ell)} & v_{13}^{II} e^{-\xi_3^{II}(\ell)} & V_{11}^{II} e^{\xi_1^{II}(\ell)} & V_{12}^{II} e^{\xi_2^{II}(\ell)} & V_{13}^{II} e^{\xi_3^{II}(\ell)} \\ v_{21}^{II} e^{-\xi_1^{II}(\ell)} & v_{22}^{II} e^{-\xi_2^{II}(\ell)} & v_{23}^{II} e^{-\xi_3^{II}(\ell)} & V_{21}^{II} e^{\xi_1^{II}(\ell)} & V_{22}^{II} e^{\xi_2^{II}(\ell)} & V_{23}^{II} e^{\xi_3^{II}(\ell)} \\ v_{41}^{II} e^{-\xi_1^{II}(-\ell)} & v_{42}^{II} e^{-\xi_2^{II}(-\ell)} & v_{43}^{II} e^{-\xi_3^{II}(-\ell)} & V_{41}^{II} e^{\xi_1^{II}(-\ell)} & V_{42}^{II} e^{\xi_2^{II}(-\ell)} & V_{43}^{II} e^{\xi_3^{II}(-\ell)} \\ v_{51}^{II} e^{-\xi_1^{II}(-\ell)} & v_{52}^{II} e^{-\xi_2^{II}(-\ell)} & v_{53}^{II} e^{-\xi_3^{II}(-\ell)} & V_{51}^{II} e^{\xi_1^{II}(-\ell)} & V_{52}^{II} e^{\xi_2^{II}(-\ell)} & V_{53}^{II} e^{\xi_3^{II}(-\ell)} \end{pmatrix},$$

$$\Lambda_3 = \begin{pmatrix} 0 & 0 & 0 & 0 & 0 & 0 \\ 0 & 0 & 0 & 0 & 0 & 0 \\ v_{11}^{III} e^{-\xi_1^{III}(\ell)} & v_{12}^{III} e^{-\xi_2^{III}(\ell)} & v_{13}^{III} e^{-\xi_3^{III}(\ell)} & V_{11}^{III} e^{\xi_1^{III}(\ell)} & V_{12}^{III} e^{\xi_2^{III}(\ell)} & V_{13}^{III} e^{\xi_3^{II}(\ell)} \\ v_{21}^{III} e^{-\xi_1^{III}(\ell)} & v_{22}^{III} e^{-\xi_2^{III}(\ell)} & v_{23}^{III} e^{-\xi_3^{III}(\ell)} & V_{21}^{III} e^{\xi_1^{III}(\ell)} & V_{22}^{III} e^{\xi_2^{III}(\ell)} & V_{23}^{III} e^{\xi_3^{III}(\ell)} \\ 0 & 0 & 0 & 0 & 0 & 0 \\ 0 & 0 & 0 & 0 & 0 & 0 \end{pmatrix},$$

$$\Lambda_4 = \begin{pmatrix} 0 & 0 & 0 & 0 & 0 & 0 \\ 0 & 0 & 0 & 0 & 0 & 0 \\ K^I v_{31}^I e^{\xi_1^I \ell} & K^I v_{32}^I e^{\xi_2^I \ell} & K^I v_{33}^I e^{\xi_3^I \ell} & K^I V_{31}^I e^{-\xi_1^I \ell} & K^I V_{32}^I e^{-\xi_2^I \ell} & K^I V_{33}^I e^{-\xi_3^I \ell} \\ -\xi_1^I K^I v_{31}^I e^{\xi_1^I \ell} & -\xi_1^I K^I v_{32}^I e^{\xi_2^I \ell} & -\xi_1^I K^I v_{33}^I e^{\xi_3^I \ell} & \xi_1^I K^I V_{31}^I e^{-\xi_1^I \ell} & \xi_1^I K^I V_{32}^I K^I e^{-\xi_2^I \ell} & \xi_1^I K^I V_{33}^I e^{-\xi_3^I \ell} \\ 0 & 0 & 0 & 0 & 0 & 0 \\ 0 & 0 & 0 & 0 & 0 & 0 \end{pmatrix},$$

$$\Lambda_5 = \begin{pmatrix} v_{41}^{II} e^{-\xi_1^{II}(\ell)} & v_{42}^{II} e^{-\xi_2^{II}(\ell)} & v_{43}^{II} e^{-\xi_3^{II}(\ell)} & V_{41}^{II} e^{\xi_1^{II}(\ell)} & V_{42}^{II} e^{\xi_2^{II}(\ell)} & V_{43}^{II} e^{\xi_3^{II}(\ell)} \\ v_{51}^{II} e^{-\xi_1^{II}(\ell)} & v_{52}^{II} e^{-\xi_2^{II}(\ell)} & v_{53}^{II} e^{-\xi_3^{II}(\ell)} & V_{51}^{II} e^{\xi_1^{II}(\ell)} & V_{52}^{II} e^{\xi_2^{II}(\ell)} & V_{53}^{II} e^{\xi_3^{II}(\ell)} \\ K^{II} v_{31}^{II} e^{\xi_1^{II}\ell} & K^{II} v_{32}^{II} e^{\xi_2^{II}\ell} & K^{II} v_{33}^{II} e^{\xi_3^{II}\ell} & K^{II} V_{31}^{II} e^{-\xi_1^{II}\ell} & K^{II} V_{32}^{II} e^{-\xi_2^{II}\ell} & K^{II} V_{33}^{II} e^{-\xi_3^{II}\ell} \\ -\xi_1^{II} K^{II} v_{31}^{II} e^{\xi_1^{II}\ell} & -\xi_1^{II} K^{II} v_{32}^{II} e^{\xi_2^{II}\ell} & -\xi_1^{II} K^{II} v_{33}^{II} e^{\xi_3^{II}\ell} & \xi_1^{II} K^{II} V_{31}^{II} e^{-\xi_1^{II}\ell} & \xi_1^{II} K^{II} V_{32}^{II} e^{-\xi_2^{II}\ell} & \xi_1^{II} K^{II} V_{33}^{II} e^{-\xi_3^{I}\ell} \\ K^{II} v_{31}^{II} e^{-\xi_1^{II}\ell} & K^{II} v_{32}^{II} e^{-\xi_2^{II}\ell} & K^{II} v_{33}^{II} e^{-\xi_3^{II}\ell} & K^{II} V_{31}^{II} e^{\xi_1^{II}\ell} & K^{II} V_{32}^{II} e^{\xi_2^{II}\ell} & K^{II} V_{33}^{II} e^{\xi_3^{II}\ell} \\ -\xi_1^{II} K^{II} v_{31}^{II} e^{-\xi_1^{II}\ell} & -\xi_2^{II} K^{II} v_{32}^{II} e^{-\xi_2^{II}\ell} & -\xi_3^{II} K^{II} v_{33}^{II} e^{-\xi_3^{II}\ell} & \xi_1^{II} K^{II} V_{31}^{II} e^{\xi_1^{II}\ell} & \xi_2^{II} K^{II} V_{32}^{II} e^{\xi_2^{II}\ell} & \xi_3^{II} K^{II} V_{33}^{II} e^{\xi_3^{II}\ell} \end{pmatrix},$$

$$\Lambda_6 = \begin{pmatrix} v_{41}^{III} e^{-\xi_1^{III}(\ell)} & v_{42}^{III} e^{-\xi_2^{III}(\ell)} & v_{43}^{III} e^{-\xi_3^{III}(\ell)} & V_{41}^{III} e^{\xi_1^{III}(\ell)} & V_{42}^{III} e^{\xi_2^{III}(\ell)} & V_{43}^{III} e^{\xi_3^{III}(\ell)} \\ v_{51}^{III} e^{-\xi_1^{III}(\ell)} & v_{52}^{III} e^{-\xi_2^{III}(\ell)} & v_{53}^{III} e^{-\xi_3^{III}(\ell)} & V_{51}^{III} e^{\xi_1^{III}(\ell)} & V_{52}^{III} e^{\xi_2^{III}(\ell)} & V_{53}^{III} e^{\xi_3^{III}(\ell)} \\ 0 & 0 & 0 & 0 & 0 & 0 \\ 0 & 0 & 0 & 0 & 0 & 0 \\ K^{III} v_{31}^{III} e^{-\xi_1^{III}\ell} & K^{III} v_{32}^{III} e^{-\xi_2^{III}\ell} & K^{III} v_{33}^{III} e^{-\xi_3^{III}\ell} & K^{III} V_{31}^{III} e^{\xi_1^{III}\ell} & K^{III} V_{32}^{III} e^{\xi_2^{III}\ell} & K^{III} V_{33}^{III} e^{\xi_3^{III}\ell} \\ -\xi_1^{III} K^{III} v_{31}^{III} e^{-\xi_1^{III}\ell} & -\xi_2^{II} K^{III} v_{32}^{II} e^{-\xi_2^{III}\ell} & -\xi_3^{III} K^{III} v_{33}^{III} e^{-\xi_3^{III}\ell} & \xi_1^{III} K^{III} V_{31}^{III} e^{\xi_1^{III}\ell} & \xi_2^{III} K^{III} V_{32}^{III} e^{\xi_2^{III}\ell} & \xi_3^{III} K^{III} V_{33}^{III} e^{\xi_3^{III}\ell} \end{pmatrix},$$

$$\Lambda_7 = \begin{pmatrix} v_{31}^I e^{-\xi_1^I(-2\ell)} & v_{32}^I e^{-\xi_2^I(-2\ell)} & v_{33}^I e^{-\xi_3^I(-2\ell)} & V_{31}^I e^{\xi_1^I(-2\ell)} & V_{32}^I e^{\xi_2^I(-2\ell)} & V_{33}^I e^{\xi_3^I(-2\ell)} \\ 0 & 0 & 0 & 0 & 0 & 0 \\ v_{41}^I e^{-\xi_1^I(-2\ell)} & v_{42}^I e^{-\xi_2^I(-2\ell)} & v_{43}^I e^{-\xi_3^I(-2\ell)} & V_{41}^I e^{\xi_1^I(-2\ell)} & V_{42}^I e^{\xi_2^I(-2\ell)} & V_{43}^I e^{\xi_3^I(-2\ell)} \\ 0 & 0 & 0 & 0 & 0 & 0 \\ v_{51}^I e^{-\xi_1^I(-2\ell)} & v_{52}^I e^{-\xi_2^I(-2\ell)} & v_{53}^I e^{-\xi_3^I(-2\ell)} & V_{51}^I e^{\xi_1^I(-2\ell)} & V_{52}^I e^{\xi_2^I(-2\ell)} & V_{53}^I e^{\xi_3^I(-2\ell)} \\ 0 & 0 & 0 & 0 & 0 & 0 \end{pmatrix},$$

$$\Lambda_8 = \begin{pmatrix} 0 & 0 & 0 & 0 & 0 & 0 \\ 0 & 0 & 0 & 0 & 0 & 0 \\ 0 & 0 & 0 & 0 & 0 & 0 \\ 0 & 0 & 0 & 0 & 0 & 0 \\ 0 & 0 & 0 & 0 & 0 & 0 \\ 0 & 0 & 0 & 0 & 0 & 0 \end{pmatrix},$$

$$\Lambda_9 = \begin{pmatrix} 0 & 0 & 0 & 0 & 0 & 0 \\ v_{31}^{III} e^{-\xi_1^{III}(2\ell)} & v_{32}^{III} e^{-\xi_2^{III}(-2\ell)} & v_{33}^{III} e^{-\xi_3^{III}(2\ell)} & V_{31}^{III} e^{\xi_1^{III}(2\ell)} & V_{32}^{III} e^{\xi_2^{III}(2\ell)} & V_{33}^{III} e^{\xi_3^{III}(2\ell)} \\ 0 & 0 & 0 & 0 & 0 & 0 \\ v_{41}^{III} e^{-\xi_1^{III}(2\ell)} & v_{42}^{III} e^{-\xi_2^{III}(2\ell)} & v_{43}^{III} e^{-\xi_3^{III}(2\ell)} & V_{41}^{III} e^{\xi_1^{III}(2\ell)} & V_{42}^{III} e^{\xi_2^{III}(2\ell)} & V_{43}^{III} e^{\xi_3^{III}(2\ell)} \\ 0 & 0 & 0 & 0 & 0 & 0 \\ v_{51}^{III} e^{-\xi_1^{III}(2\ell)} & v_{52}^{III} e^{-\xi_2^{III}(2\ell)} & v_{53}^{III} e^{-\xi_3^{III}(2\ell)} & V_{51}^{III} e^{\xi_1^{III}(2\ell)} & V_{52}^{III} e^{\xi_2^{III}(2\ell)} & V_{53}^{III} e^{\xi_3^{III}(2\ell)} \end{pmatrix},$$

$$\Lambda_{10} = \begin{pmatrix} u_{11}^I e^{-\zeta_1^I(-\ell)} & u_{12}^I e^{-\zeta_2^I(-\ell)} & u_{13}^I e^{-\zeta_3^I(-\ell)} & U_{11}^I e^{\zeta_1^I(-\ell)} & U_{12}^I e^{\zeta_2^I(-\ell)} & U_{13}^I e^{\zeta_3^I(-\ell)} \\ u_{21}^I e^{-\zeta_1^I(-\ell)} & u_{22}^I e^{-\zeta_2^I(-\ell)} & u_{23}^I e^{-\zeta_3^I(-\ell)} & U_{21}^I e^{\zeta_1^I(-\ell)} & U_{22}^I e^{\zeta_2^I(-\ell)} & U_{23}^I e^{\zeta_3^I(-\ell)} \\ 0 & 0 & 0 & 0 & 0 & 0 \\ 0 & 0 & 0 & 0 & 0 & 0 \\ u_{41}^I e^{-\zeta_1^I(-\ell)} & u_{42}^I e^{-\zeta_2^I(-\ell)} & u_{43}^I e^{-\zeta_3^I(-\ell)} & U_{41}^I e^{\zeta_1^I(-\ell)} & U_{42}^I e^{\zeta_2^I(-\ell)} & U_{43}^I e^{\zeta_3^I(-\ell)} \\ u_{51}^I e^{-\zeta_1^I(-\ell)} & u_{52}^I e^{-\zeta_2^I(-\ell)} & u_{53}^I e^{-\zeta_3^I(-\ell)} & U_{51}^I e^{\zeta_1^I(-\ell)} & U_{52}^I e^{\zeta_2^I(-\ell)} & U_{53}^I e^{\zeta_3^I(-\ell)} \end{pmatrix},$$

$$\Lambda_{11} = \begin{pmatrix} u_{11}^{II} e^{-\zeta_1^{II}(-\ell)} & u_{12}^{II} e^{-\zeta_2^{II}(-\ell)} & u_{13}^{II} e^{-\zeta_3^{II}(-\ell)} & U_{11}^{II} e^{\zeta_1^{II}(-\ell)} & U_{12}^{II} e^{\zeta_2^{II}(-\ell)} & U_{13}^{II} e^{\zeta_3^{II}(-\ell)} \\ u_{21}^{II} e^{-\zeta_1^{II}(-\ell)} & u_{22}^{II} e^{-\zeta_2^{II}(-\ell)} & u_{23}^{II} e^{-\zeta_3^{II}(-\ell)} & U_{21}^{II} e^{\zeta_1^{II}(-\ell)} & U_{22}^{II} e^{\zeta_2^{II}(-\ell)} & U_{23}^{II} e^{\zeta_3^{II}(-\ell)} \\ u_{11}^{II} e^{-\zeta_1^{II}(\ell)} & u_{12}^{II} e^{-\zeta_2^{II}(\ell)} & u_{13}^{II} e^{-\zeta_3^{II}(\ell)} & U_{11}^{II} e^{\zeta_1^{II}(\ell)} & U_{12}^{II} e^{\zeta_2^{II}(\ell)} & U_{13}^{II} e^{\zeta_3^{II}(\ell)} \\ u_{21}^{II} e^{-\zeta_1^{II}(\ell)} & u_{22}^{II} e^{-\zeta_2^{II}(\ell)} & u_{23}^{II} e^{-\zeta_3^{II}(\ell)} & U_{21}^{II} e^{\zeta_1^{II}(\ell)} & U_{22}^{II} e^{\zeta_2^{II}(\ell)} & U_{23}^{II} e^{\zeta_3^{II}(\ell)} \\ u_{41}^{II} e^{-\zeta_1^{II}(-\ell)} & u_{42}^{II} e^{-\zeta_2^{II}(-\ell)} & u_{43}^{II} e^{-\zeta_3^{II}(-\ell)} & U_{41}^{II} e^{\zeta_1^{II}(-\ell)} & U_{42}^{II} e^{\zeta_2^{II}(-\ell)} & U_{43}^{II} e^{\zeta_3^{II}(-\ell)} \\ u_{51}^{II} e^{-\zeta_1^{II}(-\ell)} & u_{52}^{II} e^{-\zeta_2^{II}(-\ell)} & u_{53}^{II} e^{-\zeta_3^{II}(-\ell)} & U_{51}^{II} e^{\zeta_1^{II}(-\ell)} & U_{52}^{II} e^{\zeta_2^{II}(-\ell)} & U_{53}^{II} e^{\zeta_3^{II}(-\ell)} \end{pmatrix},$$

$$\Lambda_{12} = \begin{pmatrix} 0 & 0 & 0 & 0 & 0 & 0 \\ 0 & 0 & 0 & 0 & 0 & 0 \\ u_{11}^{III} e^{-\zeta_1^{III}(\ell)} & u_{12}^{III} e^{-\zeta_2^{III}(\ell)} & u_{13}^{III} e^{-\zeta_3^{III}(\ell)} & U_{11}^{III} e^{\zeta_1^{III}(\ell)} & U_{12}^{III} e^{\zeta_2^{III}(\ell)} & U_{13}^{III} e^{\zeta_3^{II}(\ell)} \\ u_{21}^{III} e^{-\zeta_1^{III}(\ell)} & u_{22}^{III} e^{-\zeta_2^{III}(\ell)} & u_{23}^{III} e^{-\zeta_3^{III}(\ell)} & U_{21}^{III} e^{\zeta_1^{III}(\ell)} & U_{22}^{III} e^{\zeta_2^{III}(\ell)} & U_{23}^{III} e^{\zeta_3^{III}(\ell)} \\ 0 & 0 & 0 & 0 & 0 & 0 \\ 0 & 0 & 0 & 0 & 0 & 0 \end{pmatrix}.$$

$$\Lambda_{13} = \begin{pmatrix} 0 & 0 & 0 & 0 & 0 & 0 \\ 0 & 0 & 0 & 0 & 0 & 0 \\ K^I u_{31}^I e^{\zeta_1^I \ell} & K^I u_{32}^I e^{\zeta_2^I \ell} & K^I u_{33}^I e^{\zeta_3^I \ell} & K^I U_{31}^I e^{-\zeta_1^I \ell} & K^I U_{32}^I e^{-\zeta_2^I \ell} & K^I U_{33}^I e^{-\zeta_3^I \ell} \\ -\zeta_1^I K^I u_{31}^I e^{\zeta_1^I \ell} & -\zeta_1^I K^I u_{32}^I e^{\zeta_2^I \ell} & -\zeta_1^I K^I u_{33}^I e^{\zeta_3^I K^I \ell} & \zeta_1^I K^I U_{31}^I e^{-\zeta_1^I \ell} & \zeta_1^I U_{32}^I e^{-\zeta_2^I \ell} & \zeta_1^I K^I U_{33}^I e^{-\zeta_3^I \ell} \\ 0 & 0 & 0 & 0 & 0 & 0 \\ 0 & 0 & 0 & 0 & 0 & 0 \end{pmatrix},$$

$$\Lambda_{14} = \begin{pmatrix} u_{41}^{II} e^{-\zeta_1^{II}(\ell)} & u_{42}^{II} e^{-\zeta_2^{II}(\ell)} & u_{43}^{II} e^{-\zeta_3^{II}(\ell)} & U_{41}^{II} e^{\zeta_1^{II}(\ell)} & U_{42}^{II} e^{\zeta_2^{II}(\ell)} & U_{43}^{II} e^{\zeta_3^{II}(\ell)} \\ u_{51}^{II} e^{-\zeta_1^{II}(\ell)} & u_{52}^{II} e^{-\zeta_2^{II}(\ell)} & u_{53}^{II} e^{-\zeta_3^{II}(\ell)} & U_{51}^{II} e^{\zeta_1^{II}(\ell)} & U_{52}^{II} e^{\zeta_2^{II}(\ell)} & U_{53}^{II} e^{\zeta_3^{II}(\ell)} \\ K^{II} u_{31}^{II} e^{\zeta_1^{II}\ell} & K^{II} u_{32}^{II} e^{\zeta_2^{II}\ell} & K^{II} u_{33}^{II} e^{\zeta_3^{II}\ell} & K^{II} U_{31}^{II} e^{-\zeta_1^{II}\ell} & K^{II} U_{32}^{II} e^{-\zeta_2^{II}\ell} & K^{II} U_{33}^{II} e^{-\zeta_3^{II}\ell} \\ -\zeta_1^{II} K^{II} u_{31}^{II} e^{\zeta_1^{II}\ell} & -\zeta_1^{II} K^{II} u_{32}^{II} e^{\zeta_2^{II}\ell} & -\zeta_1^{II} K^{II} u_{33}^{II} e^{\zeta_3^{II}\ell} & \zeta_1^{II} K^{II} U_{31}^{II} e^{-\zeta_1^{II}\ell} & \zeta_1^{II} K^{II} U_{32}^{II} e^{-\zeta_2^{II}\ell} & \zeta_1^{II} K^{II} U_{33}^{II} e^{-\zeta_3^{I}\ell} \\ K^{II} u_{31}^{II} e^{-\zeta_1^{II}\ell} & K^{II} u_{32}^{II} e^{-\zeta_2^{II}\ell} & K^{II} u_{33}^{II} e^{-\zeta_3^{II}\ell} & K^{II} U_{31}^{II} e^{\zeta_1^{II}\ell} & K^{II} U_{32}^{II} e^{\zeta_2^{II}\ell} & K^{II} U_{33}^{II} e^{\zeta_3^{II}\ell} \\ -\zeta_1^{II} K^{II} u_{31}^{II} e^{-\zeta_1^{II}\ell} & -\zeta_2^{II} K^{II} u_{32}^{II} e^{-\zeta_2^{II}\ell} & -\zeta_3^{II} K^{II} u_{33}^{II} e^{-\zeta_3^{II}\ell} & \zeta_1^{II} K^{II} U_{31}^{II} e^{\zeta_1^{II}\ell} & \zeta_2^{II} K^{II} U_{32}^{II} e^{\zeta_2^{II}\ell} & \zeta_3^{II} K^{II} U_{33}^{II} e^{\zeta_3^{II}\ell} \end{pmatrix},$$

$$\Lambda_{15} = \begin{pmatrix} u_{41}^{III} e^{-\zeta_1^{III}(\ell)} & u_{42}^{III} e^{-\zeta_2^{III}(\ell)} & u_{43}^{III} e^{-\zeta_3^{III}(\ell)} & U_{41}^{III} e^{\zeta_1^{III}(\ell)} & U_{42}^{III} e^{\zeta_2^{III}(\ell)} & U_{43}^{III} e^{\zeta_3^{III}(\ell)} \\ u_{51}^{III} e^{-\zeta_1^{III}(\ell)} & u_{52}^{III} e^{-\zeta_2^{III}(\ell)} & u_{53}^{III} e^{-\zeta_3^{III}(\ell)} & U_{51}^{III} e^{\zeta_1^{III}(\ell)} & U_{52}^{III} e^{\zeta_2^{III}(\ell)} & U_{53}^{III} e^{\zeta_3^{III}(\ell)} \\ 0 & 0 & 0 & 0 & 0 & 0 \\ 0 & 0 & 0 & 0 & 0 & 0 \\ K^{III} u_{31}^{III} e^{-\zeta_1^{III}\ell} & K^{III} u_{32}^{III} e^{-\zeta_2^{III}\ell} & K^{III} u_{33}^{III} e^{-\zeta_3^{III}\ell} & K^{III} U_{31}^{III} e^{\zeta_1^{III}\ell} & K^{III} U_{32}^{III} e^{\zeta_2^{III}\ell} & K^{III} U_{33}^{III} e^{\zeta_3^{III}\ell} \\ -\zeta_1^{III} K^{III} u_{31}^{III} e^{-\zeta_1^{III}\ell} & -\zeta_2^{II} K^{III} u_{32}^{II} e^{-\zeta_2^{III}\ell} & -\zeta_3^{III} K^{III} u_{33}^{III} e^{-\zeta_3^{III}\ell} & \zeta_1^{III} K^{III} U_{31}^{III} e^{\zeta_1^{III}\ell} & \zeta_2^{III} K^{III} U_{32}^{III} e^{\zeta_2^{III}\ell} & \zeta_3^{III} K^{III} U_{33}^{III} e^{\zeta_3^{III}\ell} \end{pmatrix},$$

$$\Lambda_{16} = \begin{pmatrix} u_{31}^{I} e^{-\zeta_1^{I}(-2\ell)} & u_{32}^{I} e^{-\zeta_2^{I}(-2\ell)} & u_{33}^{I} e^{-\zeta_3^{I}(-2\ell)} & U_{31}^{I} e^{\zeta_1^{I}(-2\ell)} & U_{32}^{I} e^{\zeta_2^{I}(-2\ell)} & U_{33}^{I} e^{\zeta_3^{I}(-2\ell)} \\ 0 & 0 & 0 & 0 & 0 & 0 \\ u_{41}^{I} e^{-\zeta_1^{I}(-2\ell)} & u_{42}^{I} e^{-\zeta_2^{I}(-2\ell)} & u_{43}^{I} e^{-\zeta_3^{I}(-2\ell)} & U_{41}^{I} e^{\zeta_1^{I}(-2\ell)} & U_{42}^{I} e^{\zeta_2^{I}(-2\ell)} & U_{43}^{I} e^{\zeta_3^{I}(-2\ell)} \\ 0 & 0 & 0 & 0 & 0 & 0 \\ u_{51}^{I} e^{-\zeta_1^{I}(-2\ell)} & u_{52}^{I} e^{-\zeta_2^{I}(-2\ell)} & u_{53}^{I} e^{-\zeta_3^{I}(-2\ell)} & U_{51}^{I} e^{\zeta_1^{I}(-2\ell)} & U_{52}^{I} e^{\zeta_2^{I}(-2\ell)} & U_{53}^{I} e^{\zeta_3^{I}(-2\ell)} \\ 0 & 0 & 0 & 0 & 0 & 0 \end{pmatrix},$$

$$\Lambda_{17} = \begin{pmatrix} 0 & 0 & 0 & 0 & 0 & 0 \\ 0 & 0 & 0 & 0 & 0 & 0 \\ 0 & 0 & 0 & 0 & 0 & 0 \\ 0 & 0 & 0 & 0 & 0 & 0 \\ 0 & 0 & 0 & 0 & 0 & 0 \\ 0 & 0 & 0 & 0 & 0 & 0 \end{pmatrix},$$

$$\Lambda_{18} = \begin{pmatrix} 0 & 0 & 0 & 0 & 0 & 0 \\ u_{31}^{III} e^{-\zeta_1^{III}(2\ell)} & u_{32}^{III} e^{-\zeta_2^{III}(-2\ell)} & u_{33}^{III} e^{-\zeta_3^{III}(2\ell)} & U_{31}^{III} e^{\zeta_1^{III}(2\ell)} & U_{32}^{III} e^{\zeta_2^{III}(2\ell)} & U_{33}^{III} e^{\zeta_3^{III}(2\ell)} \\ 0 & 0 & 0 & 0 & 0 & 0 \\ u_{41}^{III} e^{-\zeta_1^{III}(2\ell)} & u_{42}^{III} e^{-\zeta_2^{III}(2\ell)} & u_{43}^{III} e^{-\zeta_3^{III}(2\ell)} & U_{41}^{III} e^{\zeta_1^{III}(2\ell)} & U_{42}^{III} e^{\zeta_2^{III}(2\ell)} & U_{43}^{III} e^{\zeta_3^{III}(2\ell)} \\ 0 & 0 & 0 & 0 & 0 & 0 \\ u_{51}^{III} e^{-\zeta_1^{III}(2\ell)} & u_{52}^{III} e^{-\zeta_2^{III}(2\ell)} & u_{53}^{III} e^{-\zeta_3^{III}(2\ell)} & U_{51}^{III} e^{\zeta_1^{III}(2\ell)} & U_{52}^{III} e^{\zeta_2^{III}(2\ell)} & U_{53}^{III} e^{\zeta_3^{III}(2\ell)} \end{pmatrix},$$
